# Supplementary material for: Chaetomadrasins A and B, Two New Cytotoxic Cytochalasans from Desert Soil-Derived Fungus Chaetomium madrasense 375
Source: Molecules. 2019 Sep 5;24(18):3240. doi: 10.3390/molecules24183240 (PMC6767004; doi:10.3390/molecules24183240)
Supplement: Supplementary file 1 [file molecules-24-03240-s001.pdf]

molecules

SUPPLEMENTARY MATERIAL

Chaetomadrasins A and B, Two New Cytotoxic Cytochalasans from Desert Soil-Derived Fungus *Chaetomium madrasense* 375

Qing-Feng Guo [1], Zhen-Hua Yin [1], Juan-Juan Zhang [1], Wen-Yi Kang [1], Xue-Wei Wang [2], Gang Ding [3,*] and Lin Chen [1,*]

1 Henan Joint International Research Laboratory of Drug Discovery of Small Molecules, Zhengzhou Key Laboratory of Synthetic Biology of Natural Products, Huanghe Science and Technology College, Zhengzhou, Henan 450063, China; guoqf.2008@163.com (Q.-F.G.); yinzhenhua1000@126.com (Z.-H.Y.); zhangjuan8908@163.com (J.-J.Z.); Kangweny@hotmail.com (W.-Y.K.)
2 Institute of Microbiology, Chinese Academy of Science, Beijing 100101, China; wangxw@im.ac.cn
3 Institute of Medicinal Plant Development, Chinese Academy of Medical Science and Union Medical College, Beijing 100193, China
* Correspondence: gding@implad.ac.cn (G.D.); lchenchina@163.com (L.C.); Tel.: +86-371-8754-0859 (L.C.)

Table of contents

Figure S1. ^{1}H NMR spectrum of compound **1** in $CDCl_3$ (400 MHz)

Figure S2. ^{13}C NMR spectrum of compound **1** in $CDCl_3$ (100 MHz)

Figure S3. COSY spectrum of compound **1** in $CDCl_3$ (400 MHz)

Figure S4. HSQC spectrum of compound **1** in $CDCl_3$ (400 MHz)

Figure S5. HMBC spectrum of compound **1** in $CDCl_3$ (400 MHz)

Figure S6. NOESY spectrum of compound **1** in $CDCl_3$ (400 MHz)

Figure S7. HRESIMS spectrum of compound **1**

Figure S8. IR spectrum of Compound **1**

Figure S9. UV spectrum of Compound **1**

Figure S10. ^{1}H NMR spectrum of compound **2** in DMSO-d_6 (400 MHz)

Figure S11. ^{13}C NMR spectrum of compound **2** in DMSO-d_6 (100 MHz)

Figure S12. COSY spectrum of compound **2** in DMSO-d_6 (400 MHz)

Figure S13. HMQC spectrum of compound **2** in DMSO-d_6 (400 MHz)

Figure S14. HMBC spectrum of compound **2** in DMSO-d_6 (400 MHz)

Figure S15. NOESY spectrum of compound **2** in DMSO-d_6 (400 MHz)

Figure S16. HRESIMS spectrum of compound **2**

Figure S17. IR spectrum of Compound **2**

Figure S18. UV spectrum of Compound **2**

Figure S19. Experimental ECD spectra of **1** and calculated ECD spectra for (3*S*, 4*R*, 5*S*, 6*S*, 8*R*, 9*R*, 16*S*)-**1** and (3*S*, 4*R*, 5*S*, 6*R*, 8*R*, 9*R*, 16*S*)-**1**

Table S1. Gibbs free energies[a] and equilibrium populations[b] of low-energy conformers of (3*S*, 4*R*, 5*S*, 6*S*, 8*R*, 9*R*, 16*S*)-**1**

Figure S20. Structures and populations of the low-energy conformers of (3*S*, 4*R*, 5*S*, 6*S*, 8*R*, 9*R*, 16*S*)-**1**

Table S2. Cartesian coordinates for the low-energy reoptimized MMFF conformers of (3*S*, 4*R*, 5*S*, 6*S*, 8*R*, 9*R*, 16*S*)-**1** at B3LYP/6-311+G (d, p) level of theory in CH_3OH

Table S3. Gibbs free energies[a] and equilibrium populations[b] of low-energy conformers of (3*S*, 4*R*, 5*S*, 6*R*, 8*R*, 9*R*, 16*S*)-**1**

Figure S21. Structures and populations of the low-energy conformers of (3*S*, 4*R*, 5*S*, 6*R*, 8*R*, 9*R*, 16*S*)-**1**

Table S4. Cartesian coordinates for the low-energy reoptimized MMFF conformers of (3*S*, 4*R*, 5*S*, 6*R*, 8*R*, 9*R*, 16*S*)-**1** at B3LYP/6-311+G (d, p) level of theory in CH_3OH

Figure S22. Experimental ECD spectra of **2** and calculated ECD spectra for (3'*R*, 3*S*, 4*R*, 7*S*, 8*R*, 9*R*, 16*S*, 17*R*, 21*R*)-**2** and (3'*S*, 3*S*, 4*R*, 7*S*, 8*R*, 9*R*, 16*S*, 17*R*, 21*R*)-**2**

Table S5. Gibbs free energies[a] and equilibrium populations[b] of low-energy conformers of (3'*R*, 3*S*, 4*R*, 7*S*, 8*R*, 9*R*, 16*S*, 17*R*, 21*R*)-**2**

Figure S23. Structures and populations of the low-energy conformers of (3'*R*, 3*S*, 4*R*, 7*S*, 8*R*, 9*R*, 16*S*, 17*R*, 21*R*)-**2**

Table S6. Cartesian coordinates for the low-energy reoptimized MMFF conformers of (3'*R*, 3*S*, 4*R*, 7*S*, 8*R*, 9*R*, 16*S*, 17*R*, 21*R*)-**2** at B3LYP/6-311+G (d, p) level of theory in CH_3OH

Table S7. Gibbs free energies[a] and equilibrium populations[b] of low-energy conformers of (3'*S*, 3*S*, 4*R*, 7*S*, 8*R*, 9*R*, 16*S*, 17*R*, 21*R*)-**2**

Figure S24. Structures and populations of the low-energy conformers of (3'*S*, 3*S*, 4*R*, 7*S*, 8*R*, 9*R*, 16*S*, 17*R*, 21*R*)-**2**

Table S8. Cartesian coordinates for the low-energy reoptimized MMFF conformers of (3'*S*, 3*S*, 4*R*, 7*S*, 8*R*, 9*R*, 16*S*, 17*R*, 21*R*)-**2** at B3LYP/6-311+G (d, p) level of theory in CH_3OH

Figure S25. 18S rDNA gene sequence of *C. madrasense* 375

Figure S26. HPLC chromatogram analysis of the crude extracts from *C. madrasense* 375 and pure compounds **1** and **2**

Figure S27. ^{1}H NMR spectrum of compound **3** in DMSO-d_6 (400 MHz)

Figure S28. ^{13}C NMR spectrum of compound **3** in DMSO-d_6 (100 MHz)

Figure S29. ^{1}H NMR spectrum of compound **4** in DMSO-d_6 (400 MHz)

Figure S30. ^{13}C NMR spectrum of compound **4** in DMSO -d_6 (100 MHz)

Figure S31. ^{1}H NMR spectrum of compound **5** in CD_3OD (400 MHz)

Figure S32. ^{13}C NMR spectrum of compound **5** in CD_3OD (100 MHz)

Figure S33. ^{1}H NMR spectrum of compound **6** in CD_3OD (400 MHz)

Figure S34. ^{13}C NMR spectrum of compound **6** in CD_3OD (100 MHz)

Figure S35. ^{1}H NMR spectrum of compound **6** in C_5D_5N (400 MHz)

Figure S36. ^{13}C NMR spectrum of compound **6** in C_5D_5N (100 MHz)

Figure S37. ^{1}H NMR spectrum of compound **7** in CD_3OD (400 MHz)

Figure S38. ^{13}C NMR spectrum of compound **7** in CD_3OD (100 MHz)

Figure S39. ^{1}H NMR spectrum of compound **8** in CD_3OD (400 MHz)

Figure S40. ^{13}C NMR spectrum of compound **8** in CD_3OD (100 MHz)

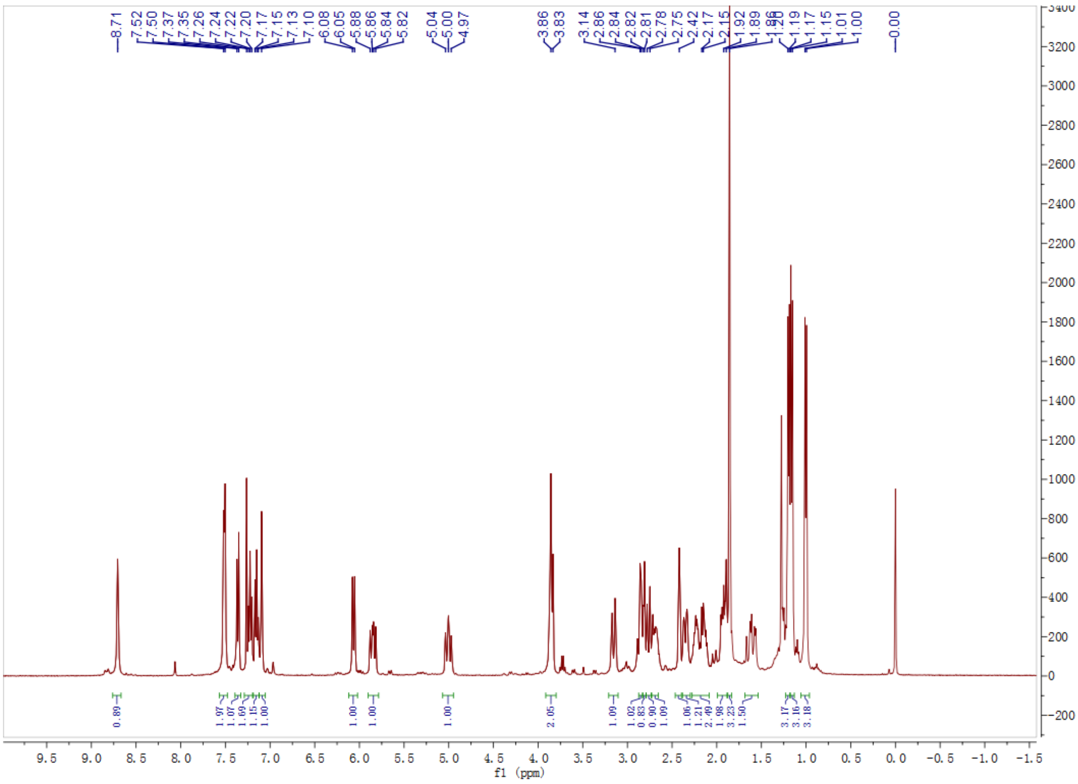

Figure S1. ^{1}H NMR spectrum of compound **1** in $CDCl_3$ (400 MHz)

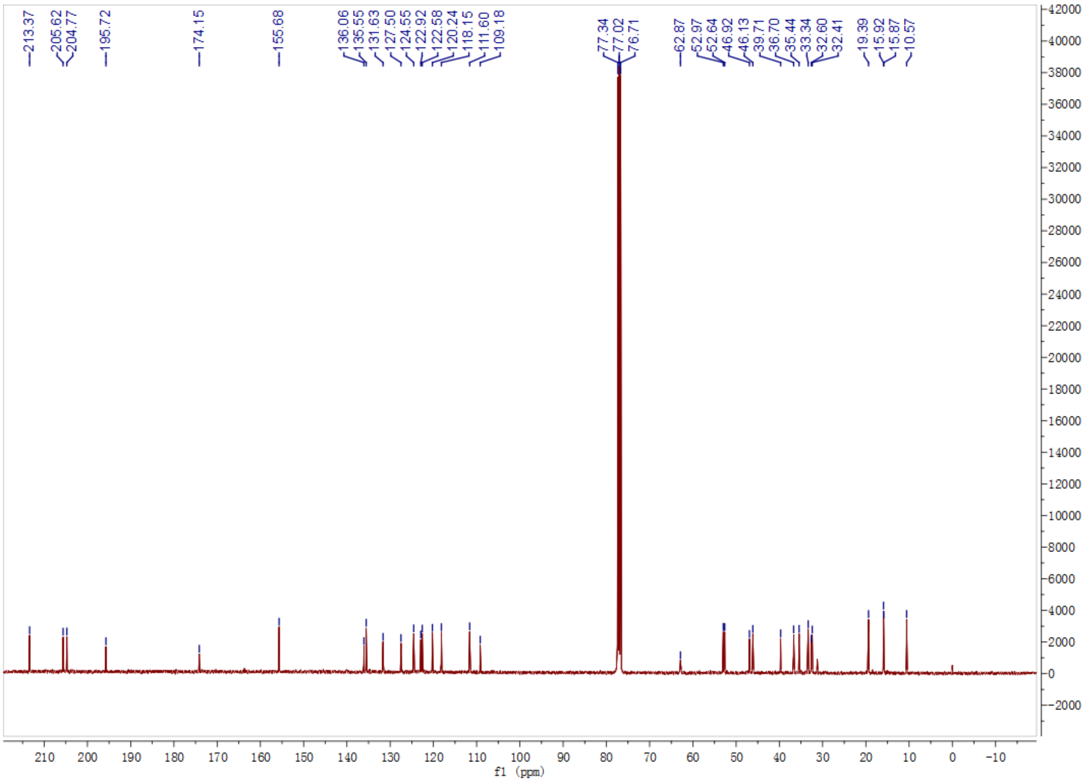

Figure S2. ^{13}C NMR spectrum of compound **1** in $CDCl_3$ (100 MHz)

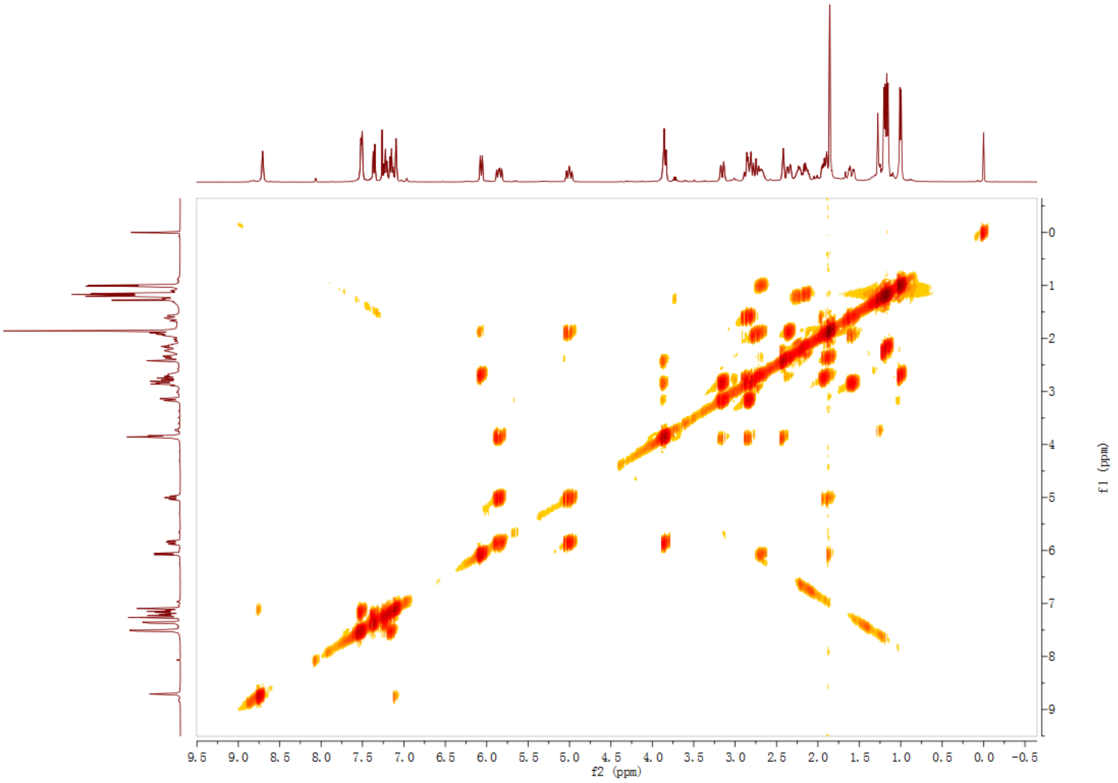

Figure S3. COSY spectrum of compound **1** in $CDCl_3$ (400 MHz)

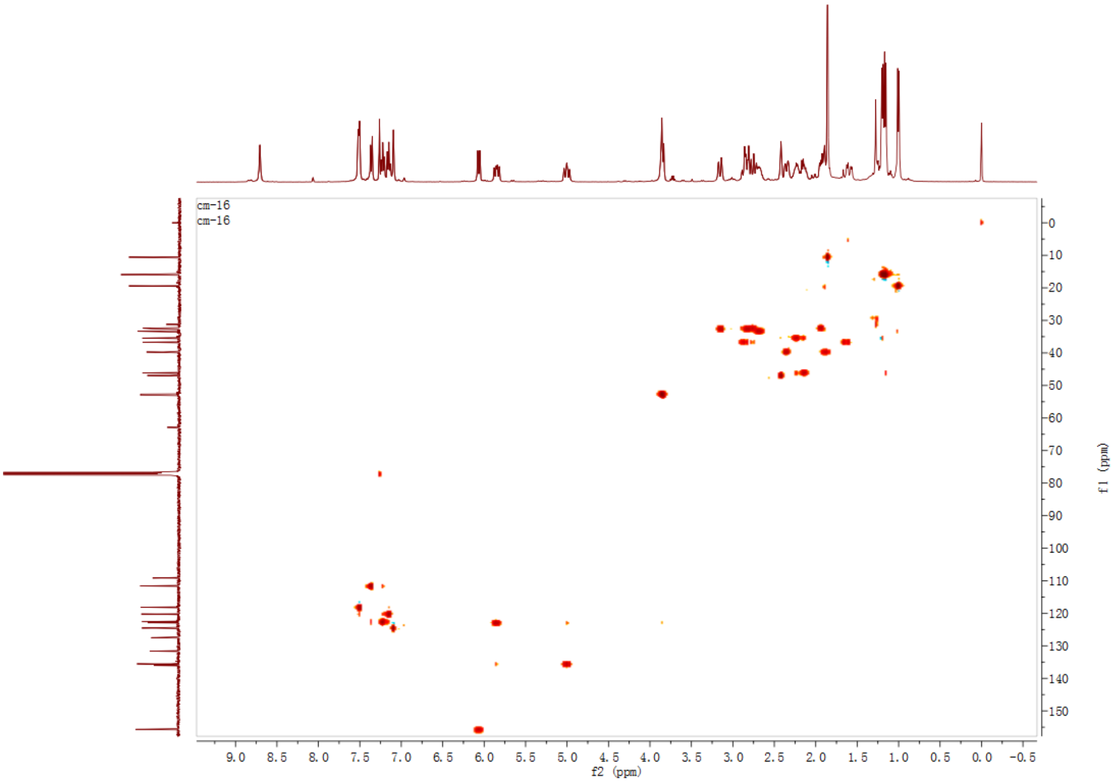

Figure S4. HSQC spectrum of compound **1** in $CDCl_3$ (400 MHz)

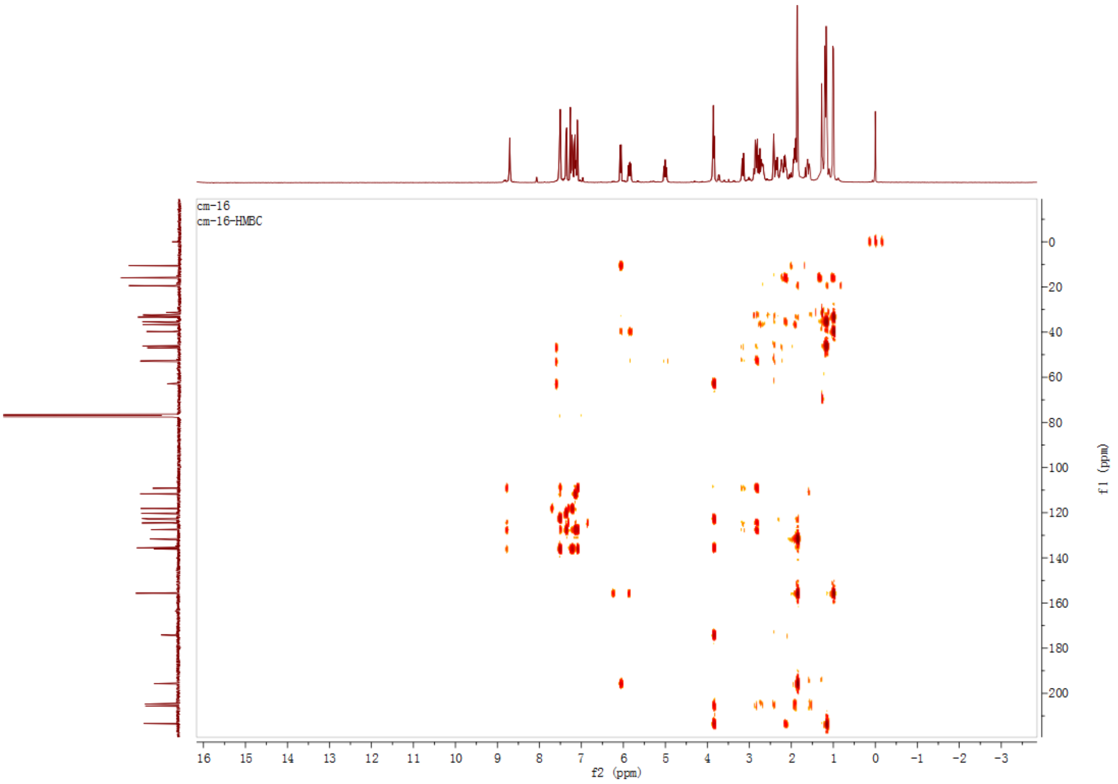

Figure S5. HMBC spectrum of compound **1** in $CDCl_3$ (400 MHz)

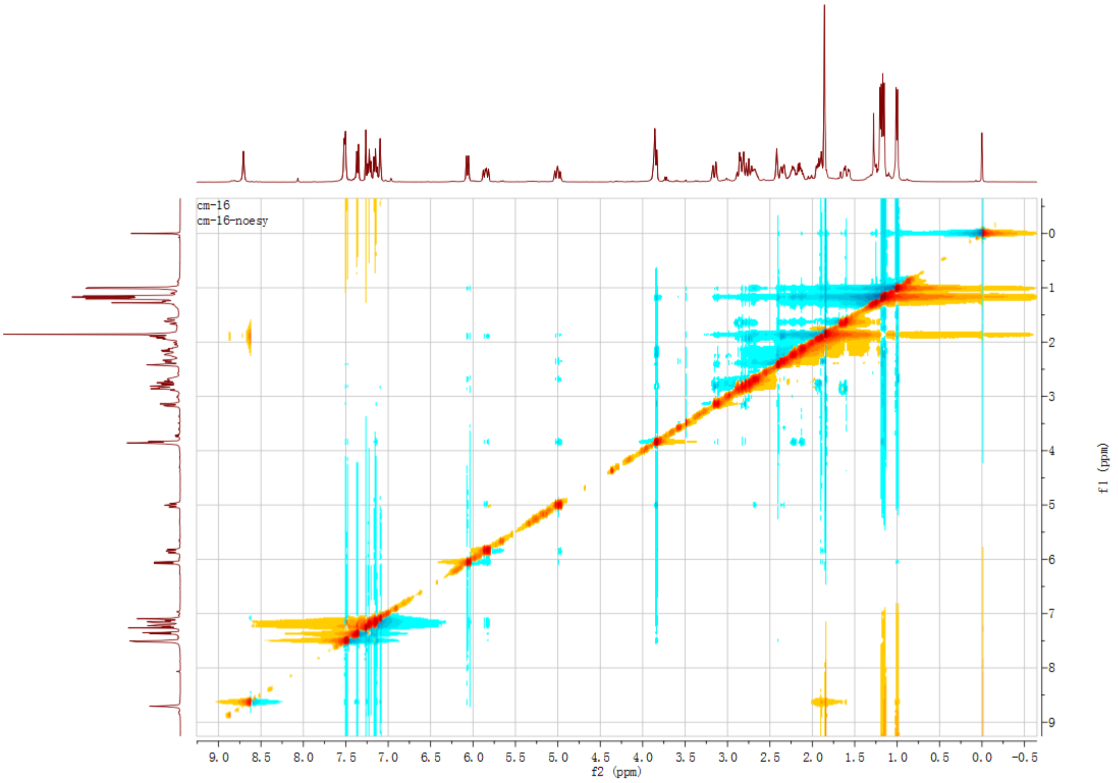

Figure S6. NOESY spectrum of compound **1** in $CDCl_3$ (400 MHz)

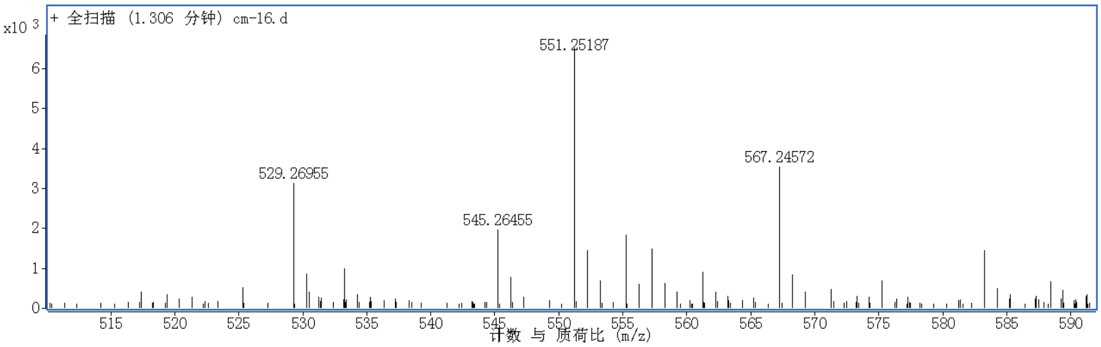

Figure S7. HRESIMS spectrum of compound **1**

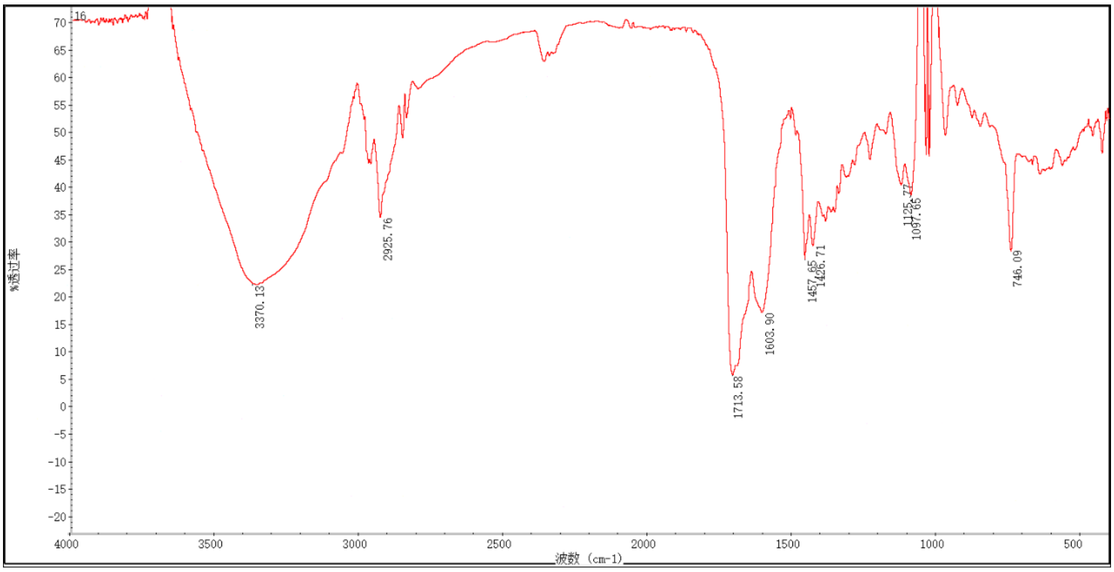

Figure S8. IR spectrum of Compound **1**

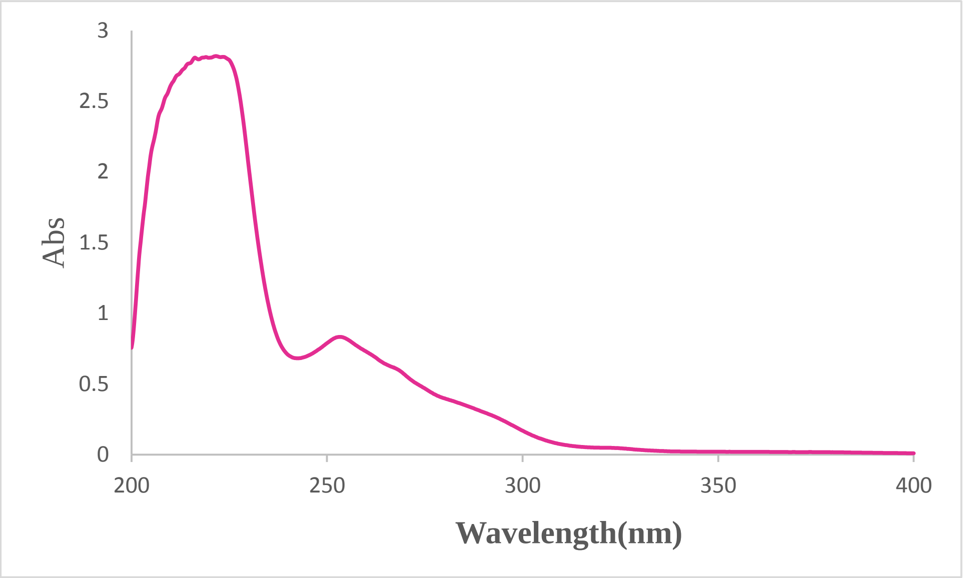

Figure S9. UV spectrum of Compound **1**

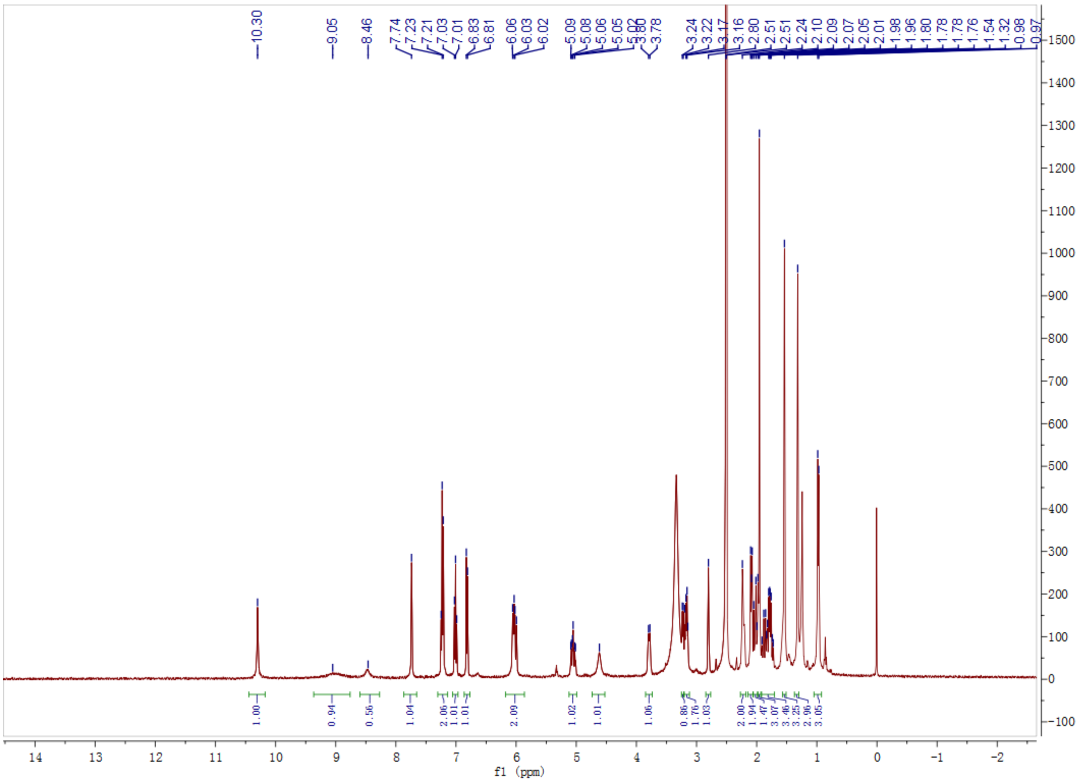

Figure S10. ^{1}H NMR spectrum of compound **2** in DMSO-d_6 (400 MHz)

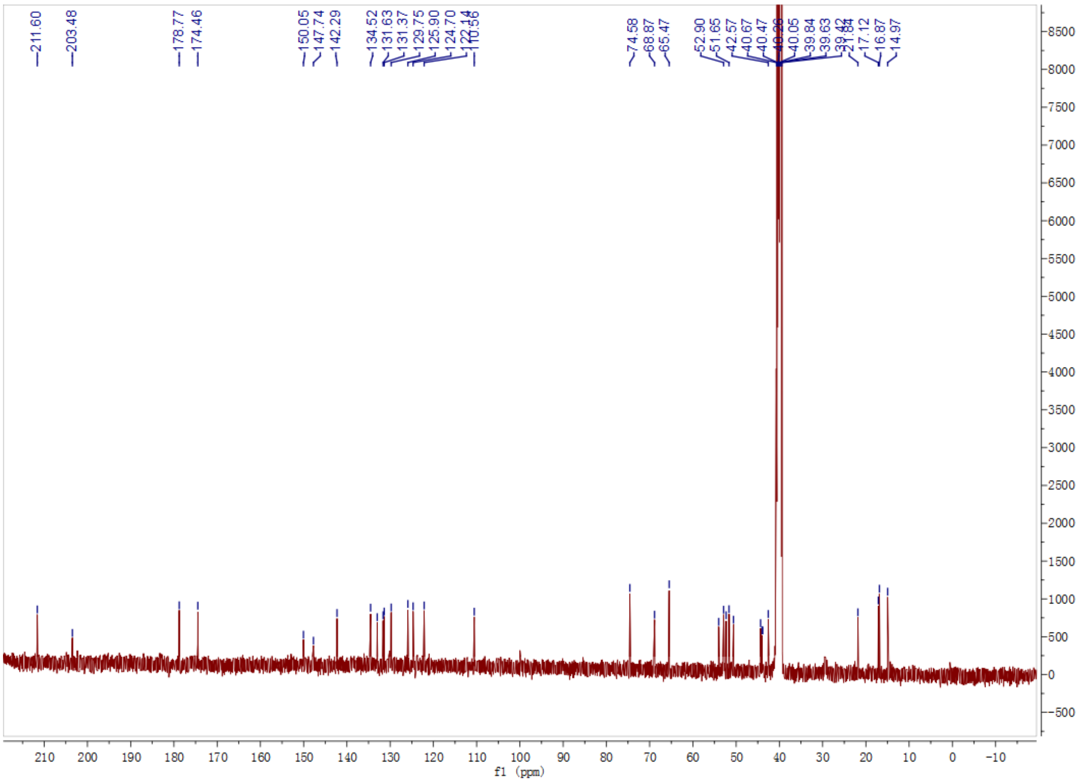

Figure S11. ^{13}C NMR spectrum of compound **2** in DMSO-d_6 (100 MHz)

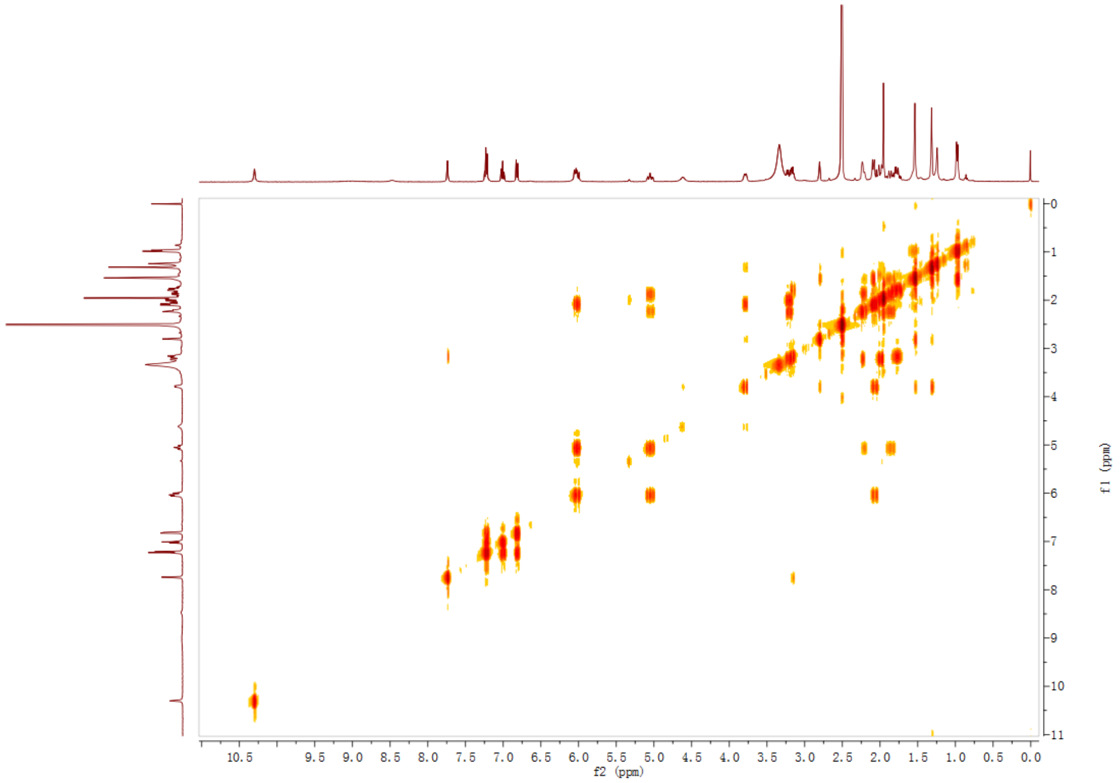

Figure S12. COSY spectrum of compound **2** in DMSO-d_6 (400 MHz)

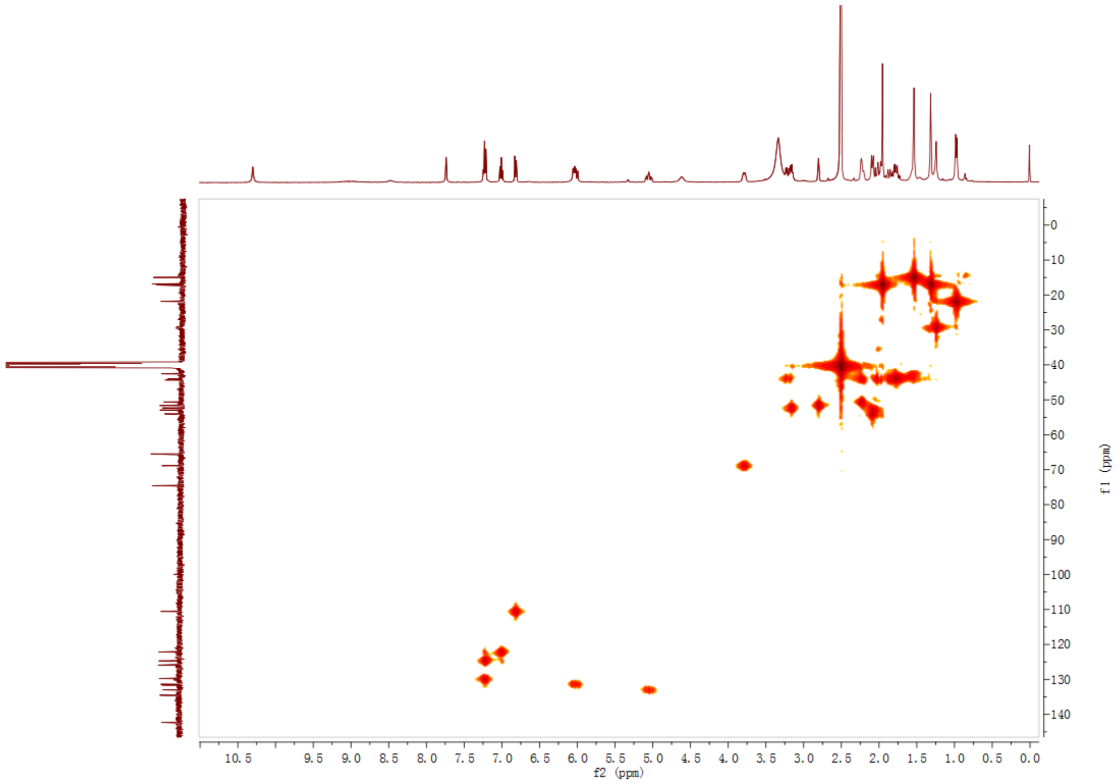

Figure S13. HMQC spectrum of compound **2** in DMSO-d_6 (400 MHz)

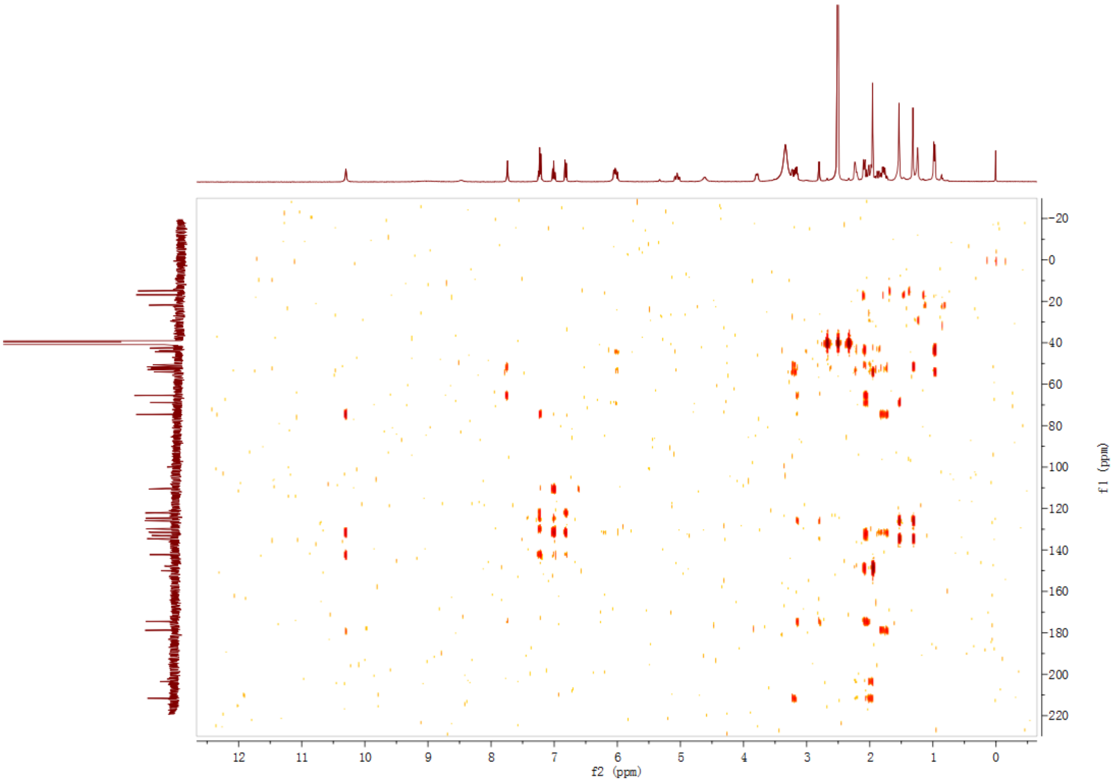

Figure S14. HMBC spectrum of compound **2** in DMSO-*d*6 (400 MHz)

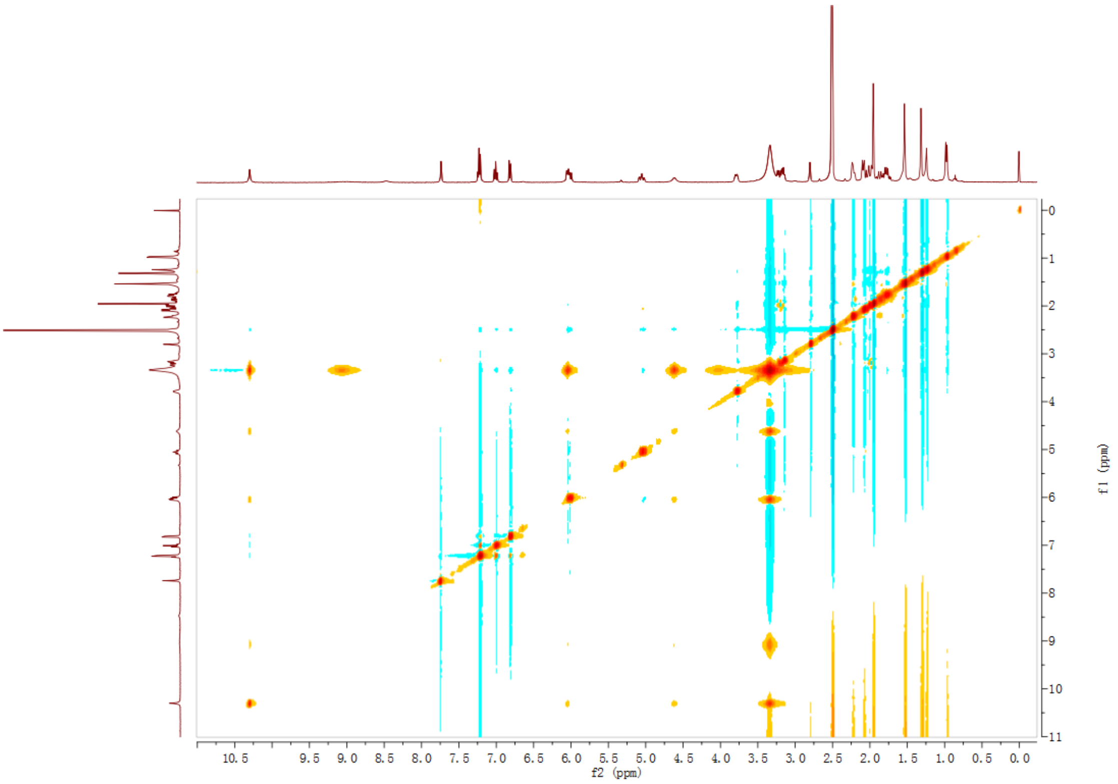

Figure S15. NOESY spectrum of compound **2** in DMSO-*d*6 (400 MHz)

CM-26: 561.25906 (M+H)$^+$, C_{32} H_{37} N_2 O_7

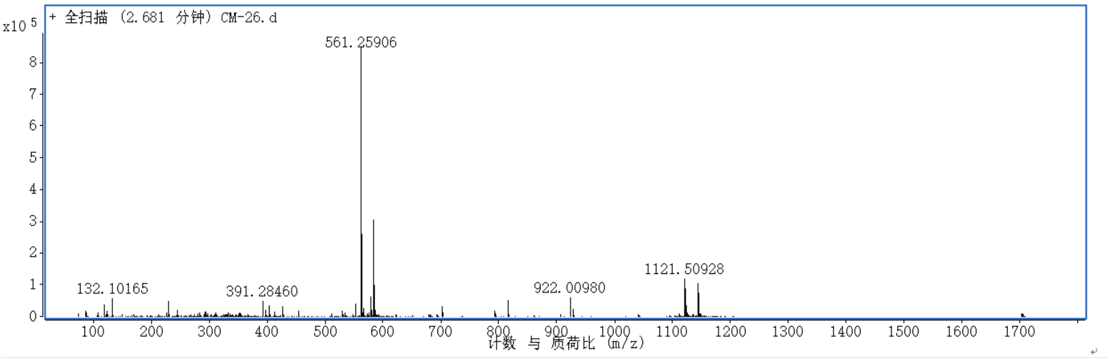

Figure S16. HRESIMS spectrum of compound **2**

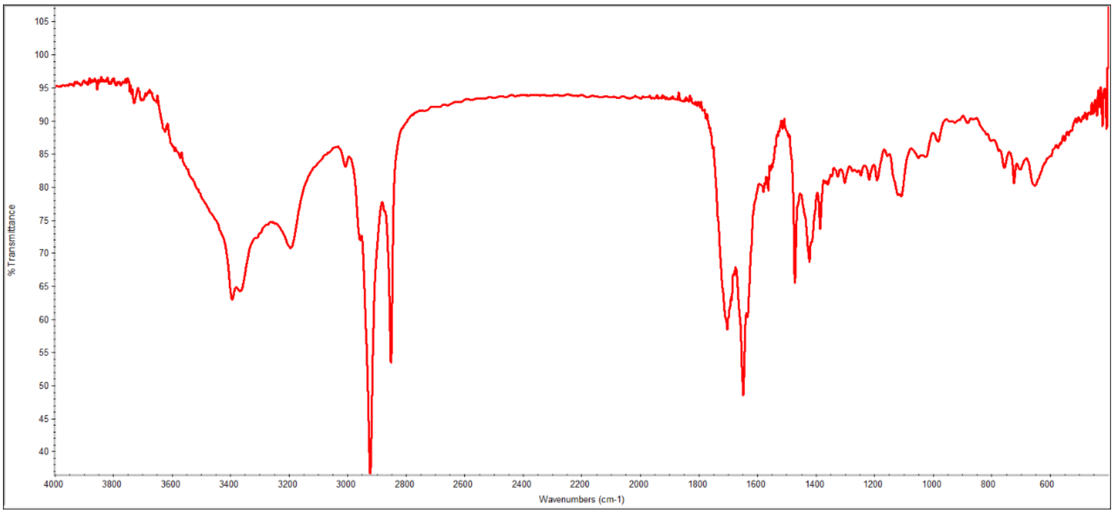

Figure S17. IR spectrum of Compound **2**

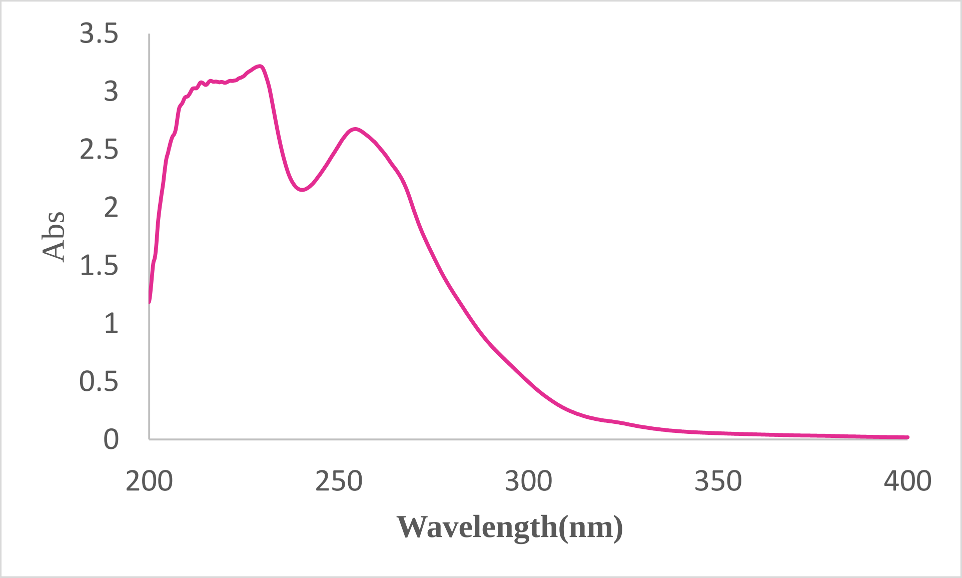

Figure S18. UV spectrum of Compound **2**

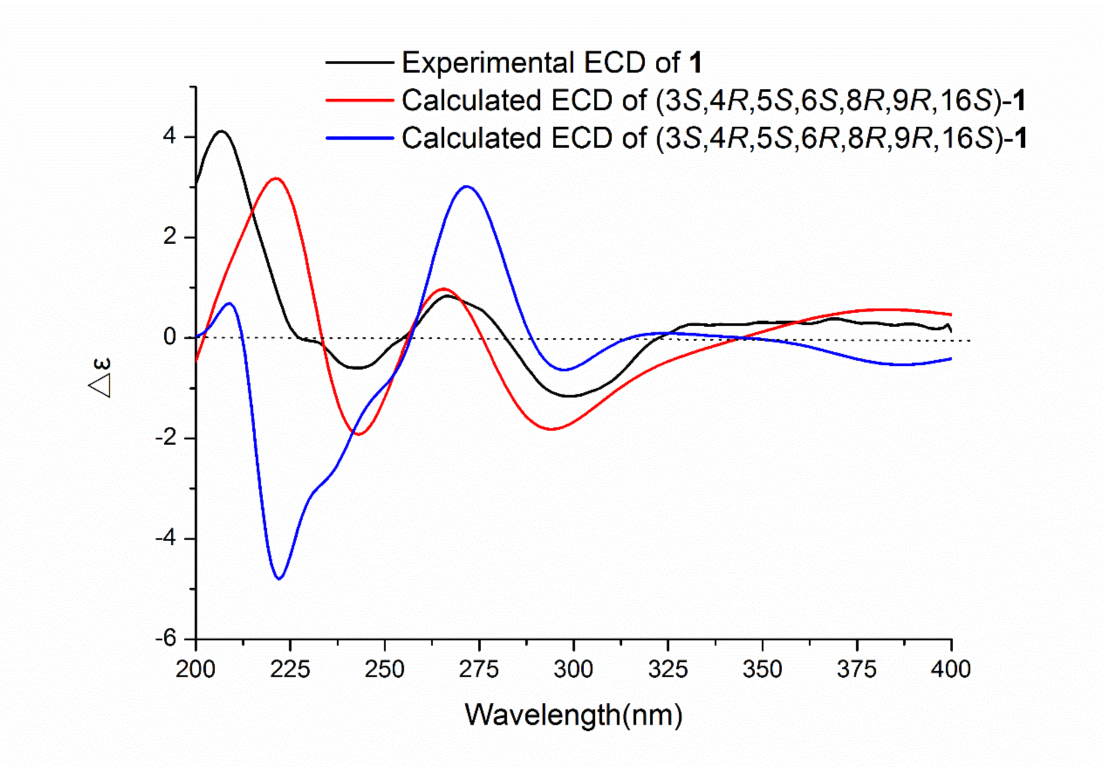

Figure S19. Experimental ECD spectra of **1** and calculated ECD spectra for (3*S*, 4*R*, 5*S*, 6*S*, 8*R*, 9*R*, 16*S*)-**1** and (3*S*, 4*R*, 5*S*, 6*R*, 8*R*, 9*R*, 16*S*)-**1**.

Table S1. Gibbs free energies[a] and equilibrium populations[b] of low-energy conformers of (3*S*, 4*R*, 5*S*, 6*S*, 8*R*, 9*R*, 16*S*)-**1**.

Conformers	In MeOH	
	ΔG	*P* (%)
**1-a**	0.00	46.2%
**1-b**	0.05	42.8%
**1-c**	1.67	2.8%
**1-d**	1.73	2.5%
**1-e**	1.89	1.9%

[a]B3LYP/6-31+G(d,p), in kcal/mol. [b]From ΔG values at 298.15K.

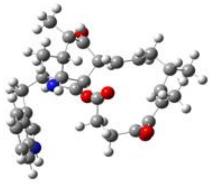

1-a 46.2%

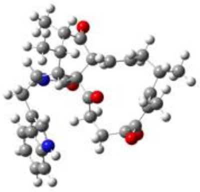

1-b 42.8%

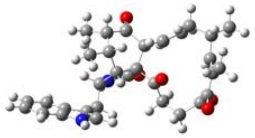

1-c 2.8%

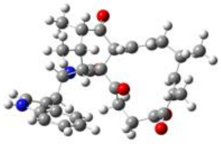

1-d 2.5%

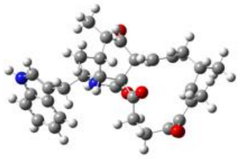

1-e 1.9%

Figure S20. Structures and populations of the low-energy conformers of (3*S*, 4*R*, 5*S*, 6*S*, 8*R*, 9*R*, 16*S*)-**1**

Table S2. Cartesian coordinates for the low-energy reoptimized MMFF conformers of (3*S*, 4*R*, 5*S*, 6*S*, 8*R*, 9*R*, 16*S*)-**1** at B3LYP/6-311+G (d, p) level of theory in CH_3OH.

**1-a**			Standard Orientation (Ångstroms)		
Center number	Atomic number	Atomic Type	X	Y	Z
1.	6.	0.	-0.952986	2.070366	1.698042
2.	6.	0.	-1.264036	1.353162	0.363117
3.	6.	0.	0.039620	1.050173	-0.474039
4.	6.	0.	1.241371	1.991805	-0.057868
5.	6.	0.	0.656996	3.375929	0.233555
6.	6.	0.	-0.199627	3.433367	1.500945
7.	6.	0.	2.386288	2.035750	-1.036013
8.	6.	0.	0.470408	-0.406949	-0.193231
9.	6.	0.	0.132446	-1.500000	-1.186739
10.	6.	0.	-5.063453	-3.246155	1.455419
11.	6.	0.	-4.694443	-3.342156	0.119736
12.	6.	0.	-4.319174	-2.162786	-0.532405
13.	6.	0.	-4.301292	-0.900186	0.121995
14.	6.	0.	-4.688826	-0.837866	1.473557
15.	6.	0.	-5.062992	-2.005290	2.125150
16.	7.	0.	-3.916962	-1.949948	-1.834612
17.	6.	0.	-3.657291	-0.605201	-2.019410
18.	6.	0.	-3.870210	0.082384	-0.846534
19.	6.	0.	-3.698317	1.558305	-0.611551
20.	8.	0.	1.044336	-0.658616	0.853281

21.	6.	0.	-0.384860	1.310987	-1.930491
22.	7.	0.	-1.605269	1.910552	-1.908049
23.	6.	0.	-2.238647	2.087832	-0.612051
24.	1.	0.	-1.729591	0.393782	0.614829
25.	8.	0.	0.256724	1.051304	-2.938854
26.	6.	0.	3.674742	1.775038	-0.775052
27.	6.	0.	4.323087	1.374634	0.525357
28.	6.	0.	5.139075	0.042545	0.485566
29.	6.	0.	4.217076	-1.107259	0.198608
30.	6.	0.	3.982200	-2.208168	0.942601
31.	6.	0.	2.892905	-3.129515	0.542197
32.	6.	0.	2.139301	-2.945390	-0.785880
33.	6.	0.	0.616770	-2.885330	-0.728132
34.	6.	0.	6.290754	0.087390	-0.539706
35.	6.	0.	4.655929	-2.572020	2.240584
36.	8.	0.	2.739057	-2.915208	-1.845554
37.	8.	0.	2.541392	-4.081062	1.229902
38.	8.	0.	0.846584	4.341694	-0.480003
39.	6.	0.	-1.023306	4.725858	1.567841
40.	6.	0.	-2.177497	2.183991	2.615316
41.	1.	0.	-0.254428	1.395906	2.206816
42.	1.	0.	1.578416	1.575962	0.896300
43.	1.	0.	0.537151	3.486873	2.318940
44.	1.	0.	2.126717	2.345408	-2.042039
45.	1.	0.	0.579337	-1.228178	-2.150077
46.	1.	0.	-0.953401	-1.492710	-1.351725
47.	1.	0.	-5.360052	-4.142005	1.992177
48.	1.	0.	-4.698898	-4.297006	-0.397964
49.	1.	0.	-4.705431	0.111415	2.001302
50.	1.	0.	-5.363402	-1.965627	3.167766
51.	1.	0.	-3.858305	-2.654746	-2.551398
52.	1.	0.	-3.339944	-0.242258	-2.986169
53.	1.	0.	-4.153409	1.828722	0.346681
54.	1.	0.	-4.250507	2.132099	-1.368233
55.	1.	0.	-2.002539	2.259674	-2.769421
56.	1.	0.	-2.287379	3.156649	-0.373505
57.	1.	0.	4.364983	1.899307	-1.608581
58.	1.	0.	3.584992	1.298789	1.329448
59.	1.	0.	5.024776	2.169715	0.818037
60.	1.	0.	5.582745	-0.073903	1.479949
61.	1.	0.	3.658564	-0.993995	-0.725944
62.	1.	0.	0.258784	-3.118444	0.275455
63.	1.	0.	0.234492	-3.637551	-1.426018
64.	1.	0.	6.922283	0.967198	-0.373588

65.	1.	0.	5.917029	0.125676	-1.567310
66.	1.	0.	6.918314	-0.804117	-0.451747
67.	1.	0.	5.111159	-3.565046	2.166217
68.	1.	0.	5.430932	-1.857528	2.521370
69.	1.	0.	3.921229	-2.632020	3.050101
70.	1.	0.	-1.494448	4.844607	2.546579
71.	1.	0.	-0.367071	5.580628	1.392215
72.	1.	0.	-1.807667	4.766871	0.806849
73.	1.	0.	-2.616864	1.196440	2.788085
74.	1.	0.	-2.954540	2.833464	2.200441
75.	1.	0.	-1.895220	2.595371	3.589701

**1-b**			Standard Orientation (Ångstroms)		
Center number	Atom number	Type	X	Y	Z
1.	6.	0.	-0.266392	3.009354	1.640306
2.	6.	0.	-0.971600	1.811741	0.958474
3.	6.	0.	-0.134319	1.184800	-0.222066
4.	6.	0.	1.233594	1.942229	-0.452922
5.	6.	0.	0.936665	3.443555	-0.564320
6.	6.	0.	0.255192	4.087242	0.650092
7.	6.	0.	2.052243	1.455567	-1.621731
8.	6.	0.	0.232683	-0.282506	0.137364
9.	6.	0.	-0.210479	-1.419868	-0.757436
10.	6.	0.	-4.693021	-3.268457	-1.563572
11.	6.	0.	-4.049492	-3.500833	-0.354675
12.	6.	0.	-3.696241	-2.385419	0.413085
13.	6.	0.	-3.965254	-1.052599	-0.006300
14.	6.	0.	-4.624765	-0.855383	-1.233321
15.	6.	0.	-4.981759	-1.958037	-1.997046
16.	7.	0.	-3.072162	-2.307510	1.641546
17.	6.	0.	-2.948190	-0.981790	2.005508
18.	6.	0.	-3.472359	-0.169235	1.026832
19.	6.	0.	-3.547119	1.330725	1.055776
20.	8.	0.	0.869990	-0.475561	1.158457
21.	6.	0.	-1.059109	1.288509	-1.449496
22.	7.	0.	-2.241776	1.825490	-1.039828
23.	6.	0.	-2.389330	2.113639	0.376373
24.	1.	0.	-1.079671	1.048979	1.733064
25.	8.	0.	-0.797858	0.950622	-2.595007
26.	6.	0.	3.345401	1.106114	-1.597193
27.	6.	0.	4.292884	1.066043	-0.425051
28.	6.	0.	5.011227	-0.305897	-0.211485

29.	6.	0.	3.974381	-1.381968	-0.074345
30.	6.	0.	3.693154	-2.168951	0.983927
31.	6.	0.	2.488360	-3.029347	0.928859
32.	6.	0.	1.630146	-3.111165	-0.347558
33.	6.	0.	0.145516	-2.813656	-0.207646
34.	6.	0.	5.993933	-0.635761	-1.352977
35.	6.	0.	4.428998	-2.204059	2.297541
36.	8.	0.	2.119557	-3.469947	-1.403898
37.	8.	0.	2.117121	-3.717662	1.872282
38.	8.	0.	1.225871	4.092756	-1.549455
39.	6.	0.	-0.726727	5.176287	0.183696
40.	6.	0.	-1.085073	3.596674	2.798377
41.	1.	0.	0.626681	2.570714	2.103097
42.	1.	0.	1.785133	1.790147	0.481157
43.	1.	0.	1.072705	4.609157	1.173515
44.	1.	0.	1.531299	1.446640	-2.572875
45.	1.	0.	0.208720	-1.260318	-1.758054
46.	1.	0.	-1.295830	-1.340401	-0.893528
47.	1.	0.	-4.980638	-4.111061	-2.184746
48.	1.	0.	-3.832117	-4.510024	-0.017113
49.	1.	0.	-4.857684	0.146992	-1.581143
50.	1.	0.	-5.488966	-1.812737	-2.945800
51.	1.	0.	-2.764109	-3.091621	2.193347
52.	1.	0.	-2.503772	-0.717077	2.954765
53.	1.	0.	-3.591965	1.678261	2.094966
54.	1.	0.	-4.488087	1.657690	0.593687
55.	1.	0.	-2.988534	1.937417	-1.711426
56.	1.	0.	-2.630500	3.174410	0.503483
57.	1.	0.	3.786551	0.829847	-2.554367
58.	1.	0.	3.777550	1.328943	0.503572
59.	1.	0.	5.074719	1.824967	-0.575317
60.	1.	0.	5.590102	-0.214508	0.713584
61.	1.	0.	3.351974	-1.489712	-0.957407
62.	1.	0.	-0.157541	-2.898349	0.837204
63.	1.	0.	-0.390402	-3.559098	-0.802795
64.	1.	0.	6.715649	0.177047	-1.488648
65.	1.	0.	5.475297	-0.790771	-2.304129
66.	1.	0.	6.550034	-1.551268	-1.130938
67.	1.	0.	4.732364	-3.228695	2.534518
68.	1.	0.	5.317900	-1.571277	2.292404
69.	1.	0.	3.772185	-1.881520	3.112409
70.	1.	0.	-1.145171	5.727904	1.029229
71.	1.	0.	-0.194920	5.878339	-0.460843
72.	1.	0.	-1.549875	4.762627	-0.405473

73.	1.	0.	-1.316385	2.823937	3.539340
74.	1.	0.	-2.030885	4.037443	2.468887
75.	1.	0.	-0.520583	4.384139	3.308883

**1-c**			Standard Orientation (Ångstroms)		
Center number	Atom number	Type	X	Y	Z
1.	6.	0.	1.327805	1.393659	-1.337248
2.	6.	0.	1.109541	0.536002	-0.063778
3.	6.	0.	-0.281894	0.813627	0.630993
4.	6.	0.	-1.152084	1.870025	-0.157296
5.	6.	0.	-0.294929	3.120945	-0.406656
6.	6.	0.	1.035203	2.901930	-1.138902
7.	6.	0.	-2.463589	2.227605	0.494639
8.	6.	0.	-1.093524	-0.506486	0.657996
9.	6.	0.	-1.423721	-1.179512	1.977115
10.	6.	0.	7.538319	-0.828592	-0.987345
11.	6.	0.	6.688603	-1.844850	-1.407372
12.	6.	0.	5.420641	-1.909968	-0.820611
13.	6.	0.	4.991639	-0.983657	0.169534
14.	6.	0.	5.877441	0.031266	0.574523
15.	6.	0.	7.137759	0.099897	-0.005004
16.	7.	0.	4.382284	-2.795055	-1.027484
17.	6.	0.	3.327247	-2.459948	-0.201178
18.	6.	0.	3.648181	-1.354416	0.549012
19.	6.	0.	2.771282	-0.663332	1.550078
20.	8.	0.	-1.430760	-0.995844	-0.405854
21.	6.	0.	0.083454	1.329910	2.036299
22.	7.	0.	1.443299	1.348429	2.134506
23.	6.	0.	2.177789	0.686647	1.064351
24.	1.	0.	1.133840	-0.501868	-0.407111
25.	8.	0.	-0.693193	1.670134	2.917018
26.	6.	0.	-3.680181	2.165112	-0.063066
27.	6.	0.	-4.071231	1.698742	-1.442554
28.	6.	0.	-5.192959	0.609959	-1.470986
29.	6.	0.	-4.745746	-0.571010	-0.660785
30.	6.	0.	-4.549625	-1.848500	-1.046149
31.	6.	0.	-3.902479	-2.784240	-0.098296
32.	6.	0.	-3.565827	-2.349535	1.340084
33.	6.	0.	-2.127358	-2.537800	1.806679
34.	6.	0.	-6.544624	1.147369	-0.958250
35.	6.	0.	-4.821438	-2.425368	-2.410806
36.	8.	0.	-4.431098	-1.937161	2.091207

37.	8.	0.	-3.598923	-3.933221	-0.397068
38.	8.	0.	-0.648437	4.227755	-0.052030
39.	6.	0.	2.136984	3.762442	-0.494078
40.	6.	0.	2.673844	1.121217	-2.021912
41.	1.	0.	0.564332	1.032330	-2.038570
42.	1.	0.	-1.334373	1.400342	-1.129819
43.	1.	0.	0.863161	3.317145	-2.144622
44.	1.	0.	-2.379059	2.619844	1.502199
45.	1.	0.	-2.029987	-0.486995	2.573771
46.	1.	0.	-0.494290	-1.298835	2.549802
47.	1.	0.	8.529987	-0.749819	-1.422342
48.	1.	0.	6.999521	-2.561629	-2.161962
49.	1.	0.	5.585207	0.750583	1.334896
50.	1.	0.	7.829072	0.878768	0.302113
51.	1.	0.	4.400915	-3.582707	-1.654155
52.	1.	0.	2.420515	-3.049052	-0.203089
53.	1.	0.	3.342810	-0.466677	2.467828
54.	1.	0.	1.944386	-1.322475	1.842200
55.	1.	0.	1.864390	1.584357	3.023444
56.	1.	0.	3.003889	1.322868	0.734879
57.	1.	0.	-4.506660	2.518980	0.552483
58.	1.	0.	-3.204660	1.309765	-1.985601
59.	1.	0.	-4.440780	2.561686	-2.015832
60.	1.	0.	-5.322920	0.323625	-2.520004
61.	1.	0.	-4.516451	-0.318771	0.369882
62.	1.	0.	-1.582527	-3.175343	1.108362
63.	1.	0.	-2.168290	-3.029025	2.783960
64.	1.	0.	-6.836707	2.046607	-1.511414
65.	1.	0.	-6.505139	1.401223	0.105485
66.	1.	0.	-7.330941	0.397798	-1.086462
67.	1.	0.	-5.476989	-3.298319	-2.329464
68.	1.	0.	-5.289776	-1.701941	-3.079887
69.	1.	0.	-3.892646	-2.780044	-2.869825
70.	1.	0.	3.064431	3.719884	-1.070621
71.	1.	0.	1.797788	4.799339	-0.455750
72.	1.	0.	2.348088	3.452214	0.532788
73.	1.	0.	2.775278	0.059689	-2.266217
74.	1.	0.	3.533451	1.395339	-1.405128
75.	1.	0.	2.744201	1.689359	-2.955744

**1-d**			Standard Orientation (Ångstroms)		
Center	Atom	Type	X	Y	Z

number	number				
1.	6.	0.	1.441779	1.772228	-1.445881
2.	6.	0.	1.301686	1.066960	-0.071882
3.	6.	0.	-0.135445	1.231399	0.560752
4.	6.	0.	-1.139309	1.975099	-0.403787
5.	6.	0.	-0.501658	3.302909	-0.848728
6.	6.	0.	0.897388	3.220442	-1.474478
7.	6.	0.	-2.519870	2.197949	0.160461
8.	6.	0.	-0.725415	-0.179527	0.828323
9.	6.	0.	-1.073276	-0.611890	2.240736
10.	6.	0.	4.376860	-3.701452	-1.628433
11.	6.	0.	5.436866	-2.833187	-1.399966
12.	6.	0.	5.210635	-1.749196	-0.545087
13.	6.	0.	3.953493	-1.517488	0.080076
14.	6.	0.	2.901940	-2.419596	-0.171560
15.	6.	0.	3.121956	-3.496997	-1.019158
16.	7.	0.	6.062451	-0.744963	-0.133997
17.	6.	0.	5.390013	0.097004	0.728905
18.	6.	0.	4.092575	-0.327641	0.889619
19.	6.	0.	3.046067	0.341071	1.736858
20.	8.	0.	-0.873832	-0.928627	-0.121264
21.	6.	0.	0.100782	2.031916	1.855979
22.	7.	0.	1.443987	2.220468	1.989331
23.	6.	0.	2.298305	1.512335	1.045179
24.	1.	0.	1.473853	0.009308	-0.278083
25.	8.	0.	-0.748992	2.429771	2.639929
26.	6.	0.	-3.682769	1.844809	-0.403243
27.	6.	0.	-3.925110	1.116877	-1.701108
28.	6.	0.	-4.846202	-0.141235	-1.581822
29.	6.	0.	-4.262578	-1.073119	-0.560843
30.	6.	0.	-3.777400	-2.321201	-0.720559
31.	6.	0.	-3.066997	-2.959575	0.411701
32.	6.	0.	-2.940241	-2.248153	1.771628
33.	6.	0.	-1.536790	-2.077173	2.338267
34.	6.	0.	-6.298902	0.228004	-1.219986
35.	6.	0.	-3.785934	-3.131008	-1.990476
36.	8.	0.	-3.927599	-1.900610	2.394326
37.	8.	0.	-2.545269	-4.065176	0.333520
38.	8.	0.	-1.074458	4.366508	-0.722523
39.	6.	0.	1.796949	4.327931	-0.895104
40.	6.	0.	2.845947	1.635589	-2.049900
41.	1.	0.	0.783635	1.196573	-2.109773
42.	1.	0.	-1.197479	1.330489	-1.287934
43.	1.	0.	0.735764	3.472508	-2.534249

44.	1.	0.	-2.544758	2.746701	1.095723
45.	1.	0.	-1.830377	0.075737	2.636922
46.	1.	0.	-0.193401	-0.450197	2.877338
47.	1.	0.	4.517744	-4.553545	-2.286408
48.	1.	0.	6.404964	-2.991302	-1.866714
49.	1.	0.	1.925845	-2.281830	0.284546
50.	1.	0.	2.314298	-4.194714	-1.217228
51.	1.	0.	7.032035	-0.660486	-0.391003
52.	1.	0.	5.897828	0.940343	1.176536
53.	1.	0.	3.514056	0.739821	2.646408
54.	1.	0.	2.307931	-0.396197	2.076211
55.	1.	0.	1.795935	2.656996	2.831009
56.	1.	0.	3.051465	2.198231	0.645312
57.	1.	0.	-4.584395	2.138661	0.133266
58.	1.	0.	-2.980833	0.805461	-2.157740
59.	1.	0.	-4.404339	1.807392	-2.410657
60.	1.	0.	-4.856506	-0.614824	-2.569009
61.	1.	0.	-4.198018	-0.633482	0.429797
62.	1.	0.	-0.838978	-2.742011	1.825918
63.	1.	0.	-1.583558	-2.351607	3.396602
64.	1.	0.	-6.703430	0.951547	-1.935947
65.	1.	0.	-6.370819	0.664919	-0.219196
66.	1.	0.	-6.937783	-0.659865	-1.235258
67.	1.	0.	-4.241111	-4.110163	-1.811696
68.	1.	0.	-4.332479	-2.634835	-2.793842
69.	1.	0.	-2.762336	-3.324316	-2.328577
70.	1.	0.	2.752779	4.378492	-1.422956
71.	1.	0.	1.288976	5.288288	-1.003335
72.	1.	0.	1.992801	4.179468	0.170298
73.	1.	0.	3.132763	0.582688	-2.125181
74.	1.	0.	3.614904	2.143733	-1.461442
75.	1.	0.	2.867076	2.064039	-3.057794

**1-e**			Standard Orientation (Ångstroms)		
Center number	Atom number	Type	X	Y	Z
1.	6.	0.	-0.320133	2.227276	1.692819
2.	6.	0.	-0.750550	0.853158	1.127178
3.	6.	0.	0.230060	0.328694	0.009488
4.	6.	0.	1.041458	1.506400	-0.664728
5.	6.	0.	0.105620	2.713332	-0.763990
6.	6.	0.	-0.244607	3.340793	0.589240
7.	6.	0.	1.692455	1.160782	-1.978667

8.	6.	0.	1.251136	-0.626311	0.668117
9.	6.	0.	1.057326	-2.126816	0.565897
10.	6.	0.	-6.721861	-2.745424	-1.479315
11.	6.	0.	-7.266168	-1.536368	-1.064233
12.	6.	0.	-6.460293	-0.698192	-0.286807
13.	6.	0.	-5.129112	-1.043573	0.078535
14.	6.	0.	-4.610460	-2.279000	-0.354509
15.	6.	0.	-5.407723	-3.114014	-1.126386
16.	7.	0.	-6.727394	0.536565	0.266780
17.	6.	0.	-5.618901	0.971920	0.965251
18.	6.	0.	-4.610093	0.042832	0.879247
19.	6.	0.	-3.235542	0.164535	1.475873
20.	8.	0.	2.182388	-0.152134	1.297680
21.	6.	0.	-0.702009	-0.367546	-1.002163
22.	7.	0.	-1.984448	-0.080892	-0.657174
23.	6.	0.	-2.182625	0.760225	0.512278
24.	1.	0.	-0.718099	0.141239	1.962755
25.	8.	0.	-0.355899	-1.057042	-1.951337
26.	6.	0.	2.990400	1.287232	-2.287632
27.	6.	0.	4.129248	1.790283	-1.438010
28.	6.	0.	5.331402	0.804770	-1.274839
29.	6.	0.	4.883065	-0.420514	-0.531893
30.	6.	0.	5.338189	-0.911669	0.639808
31.	6.	0.	4.620249	-2.041007	1.276151
32.	6.	0.	3.476525	-2.770268	0.551071
33.	6.	0.	2.154325	-2.917150	1.297304
34.	6.	0.	5.966477	0.422506	-2.627456
35.	6.	0.	6.472938	-0.358539	1.462638
36.	8.	0.	3.646318	-3.263206	-0.549292
37.	8.	0.	4.897122	-2.457494	2.395077
38.	8.	0.	-0.326179	3.136800	-1.818086
39.	6.	0.	-1.423646	4.314808	0.468986
40.	6.	0.	-1.129361	2.653329	2.924861
41.	1.	0.	0.705367	2.062163	2.044151
42.	1.	0.	1.805040	1.754930	0.078497
43.	1.	0.	0.641042	3.947433	0.838798
44.	1.	0.	1.020093	0.806452	-2.751836
45.	1.	0.	1.028412	-2.388804	-0.498056
46.	1.	0.	0.060624	-2.377930	0.954291
47.	1.	0.	-7.319910	-3.418953	-2.085458
48.	1.	0.	-8.279626	-1.253637	-1.334145
49.	1.	0.	-3.600303	-2.577510	-0.089640
50.	1.	0.	-5.014328	-4.067314	-1.465540
51.	1.	0.	-7.599582	1.033675	0.192308

52.	1.	0.	-5.636282	1.920792	1.483461
53.	1.	0.	-2.879151	-0.819076	1.807616
54.	1.	0.	-3.276914	0.799878	2.366963
55.	1.	0.	-2.750547	-0.394370	-1.238599
56.	1.	0.	-2.538616	1.747946	0.196621
57.	1.	0.	3.265516	1.020834	-3.307371
58.	1.	0.	3.783053	2.075656	-0.440028
59.	1.	0.	4.527819	2.703543	-1.904256
60.	1.	0.	6.087550	1.339736	-0.690734
61.	1.	0.	4.060814	-0.941729	-1.013837
62.	1.	0.	2.259627	-2.589357	2.332414
63.	1.	0.	1.887404	-3.978832	1.284464
64.	1.	0.	6.227741	1.319807	-3.199324
65.	1.	0.	5.289233	-0.183745	-3.236507
66.	1.	0.	6.878942	-0.160360	-2.472381
67.	1.	0.	7.216014	-1.138892	1.656428
68.	1.	0.	6.971963	0.479633	0.974297
69.	1.	0.	6.111376	-0.030809	2.442874
70.	1.	0.	-1.550424	4.899107	1.383644
71.	1.	0.	-1.238039	5.001836	-0.358997
72.	1.	0.	-2.368876	3.808362	0.254864
73.	1.	0.	-1.103624	1.872061	3.691755
74.	1.	0.	-2.177142	2.859871	2.688359
75.	1.	0.	-0.710013	3.562358	3.367882

Table S3. Gibbs free energies[a] and equilibrium populations[b] of low-energy conformers of (3*S*, 4*R*, 5*S*, 6*R*, 8*R*, 9*R*, 16*S*)-**1**.

Conformers	In MeOH	
	Δ*G*	*P* (%)
**1-a**	0.00	25.8%
**1-b**	0.02	24.8%
**1-c**	0.21	18.2%
**1-d**	0.71	7.8%
**1-e**	0.82	6.5%

[a]B3LYP/6-31+G (d, p), in kcal/mol. [b]From Δ*G* values at 298.15K.

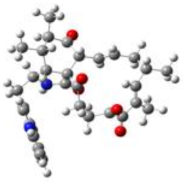

1-a 25.8%

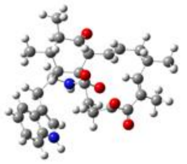

1-b 24.8%

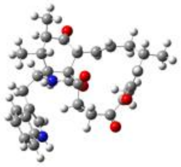

1-c 18.2%

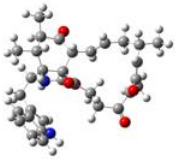

1-d 7.8%

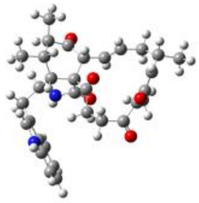

1-e 6.5%

Figure S21. Structures and populations of the low-energy conformers of (3*S*, 4*R*, 5*S*, 6*R*, 8*R*, 9*R*, 16*S*)-**1**

Table S4. Cartesian coordinates for the low-energy reoptimized MMFF conformers of (3*S*, 4*R*, 5*S*, 6*R*, 8*R*, 9*R*, 16*S*)-**1** at B3LYP/6-311+G (d, p) level of theory in CH_3OH.

**1-a**			Standard Orientation (Ångstroms)		
Center number	Atomic number	Atomic Type	X	Y	Z
1.	6.	0.	-1.365908	3.301252	1.071146
2.	6.	0.	-1.559368	1.879102	0.491582
3.	6.	0.	-0.225502	1.251263	-0.071751
4.	6.	0.	0.944671	2.302112	-0.134813
5.	6.	0.	0.404604	3.589790	-0.747974
6.	6.	0.	-0.736320	4.258468	0.031847
7.	6.	0.	2.201348	1.789205	-0.783135
8.	6.	0.	0.234124	0.107702	0.873954
9.	6.	0.	-0.019483	-1.328378	0.463229
10.	6.	0.	-3.748864	-4.219799	-1.235869
11.	6.	0.	-3.737802	-3.928024	0.121814
12.	6.	0.	-3.784661	-2.579266	0.491985
13.	6.	0.	-3.833440	-1.525011	-0.462317
14.	6.	0.	-3.850535	-1.858828	-1.829301
15.	6.	0.	-3.809246	-3.194695	-2.202344
16.	7.	0.	-3.801478	-1.997954	1.743621
17.	6.	0.	-3.867613	-0.626185	1.606295
18.	6.	0.	-3.878768	-0.281323	0.274285
19.	6.	0.	-3.952005	1.113484	-0.279051
20.	8.	0.	0.746376	0.397142	1.940236

21.	6.	0.	-0.600007	0.753908	-1.480293
22.	7.	0.	-1.895637	1.092800	-1.717677
23.	6.	0.	-2.607767	1.793178	-0.664322
24.	1.	0.	-1.915565	1.243328	1.307742
25.	8.	0.	0.129701	0.162505	-2.263473
26.	6.	0.	3.358743	1.692787	-0.124366
27.	6.	0.	4.631307	1.120917	-0.684237
28.	6.	0.	5.232293	-0.007708	0.209477
29.	6.	0.	4.172869	-1.046450	0.507884
30.	6.	0.	3.827678	-2.168202	-0.161157
31.	6.	0.	2.640811	-2.919427	0.317965
32.	6.	0.	1.911672	-2.551748	1.631971
33.	6.	0.	0.407304	-2.362083	1.513468
34.	6.	0.	6.562631	-0.521350	-0.361773
35.	6.	0.	4.469077	-2.714477	-1.416336
36.	8.	0.	2.503988	-2.581045	2.692720
37.	8.	0.	2.170510	-3.884424	-0.275952
38.	8.	0.	0.848291	4.083716	-1.765940
39.	6.	0.	-0.195819	5.563827	0.650413
40.	6.	0.	-2.665147	3.858654	1.671940
41.	1.	0.	2.125771	1.468531	-1.817111
42.	1.	0.	3.402679	2.018465	0.917168
43.	1.	0.	-0.654472	3.192869	1.900787
44.	1.	0.	1.165245	2.518542	0.918620
45.	1.	0.	-1.487988	4.537831	-0.718591
46.	1.	0.	0.474278	-1.495124	-0.501186
47.	1.	0.	-1.089548	-1.430942	0.240203
48.	1.	0.	-3.711148	-5.255486	-1.558904
49.	1.	0.	-3.696953	-4.717191	0.866943
50.	1.	0.	-3.899774	-1.083360	-2.588013
51.	1.	0.	-3.819024	-3.458612	-3.255286
52.	1.	0.	-3.798155	-2.494978	2.619192
53.	1.	0.	-3.919604	0.008184	2.480376
54.	1.	0.	-4.443792	1.770463	0.447205
55.	1.	0.	-4.595007	1.122326	-1.169313
56.	1.	0.	-2.305016	0.876066	-2.615612
57.	1.	0.	-2.865295	2.802370	-1.013193
58.	1.	0.	5.394609	1.907879	-0.773179
59.	1.	0.	4.453115	0.733126	-1.693840
60.	1.	0.	5.462181	0.460060	1.177341
61.	1.	0.	3.574244	-0.801743	1.380297
62.	1.	0.	0.031376	-2.103719	2.505371
63.	1.	0.	-0.002802	-3.334090	1.215875
64.	1.	0.	7.301150	0.287242	-0.356448

65.	1.	0.	6.467492	-0.872055	-1.391741
66.	1.	0.	6.961548	-1.342682	0.241059
67.	1.	0.	4.590641	-1.941385	-2.179384
68.	1.	0.	5.455576	-3.142092	-1.210411
69.	1.	0.	3.839150	-3.506578	-1.824510
70.	1.	0.	0.555093	5.353347	1.420520
71.	1.	0.	-0.996152	6.148729	1.111621
72.	1.	0.	0.271711	6.174372	-0.125575
73.	1.	0.	-3.088942	3.163722	2.404884
74.	1.	0.	-3.425390	4.043901	0.904560
75.	1.	0.	-2.487622	4.805425	2.188896

**1-b**			Standard Orientation (Ångstroms)		
Center number	Atom number	Type	X	Y	Z
1.	6.	0.	1.631661	2.807804	-1.085495
2.	6.	0.	1.592368	1.587254	-0.134430
3.	6.	0.	0.121326	1.133987	0.215256
4.	6.	0.	-0.948215	2.222179	-0.181884
5.	6.	0.	-0.417794	3.591637	0.222864
6.	6.	0.	0.873869	4.018304	-0.486042
7.	6.	0.	-2.338453	1.919488	0.305784
8.	6.	0.	-0.217834	-0.155127	-0.578822
9.	6.	0.	-0.030351	-1.499337	0.095557
10.	6.	0.	4.733350	-3.226796	-1.706433
11.	6.	0.	4.176924	-3.592839	-0.487386
12.	6.	0.	3.855619	-2.567904	0.409166
13.	6.	0.	4.073564	-1.194405	0.109230
14.	6.	0.	4.645334	-0.859827	-1.132448
15.	6.	0.	4.967965	-1.873232	-2.024756
16.	7.	0.	3.310385	-2.624949	1.675460
17.	6.	0.	3.185982	-1.344301	2.177026
18.	6.	0.	3.632626	-0.428197	1.252886
19.	6.	0.	3.667292	1.066764	1.408978
20.	8.	0.	-0.579040	-0.061030	-1.737805
21.	6.	0.	0.139794	0.934631	1.742274
22.	7.	0.	1.330022	1.396206	2.211889
23.	6.	0.	2.317648	1.814943	1.233688
24.	1.	0.	2.094774	0.759069	-0.644470
25.	8.	0.	-0.758243	0.471656	2.431762
26.	6.	0.	-3.357708	1.695173	-0.526971
27.	6.	0.	-4.743993	1.289441	-0.110001
28.	6.	0.	-5.228456	-0.010223	-0.824611

29.	6.	0.	-4.180588	-1.095874	-0.698658
30.	6.	0.	-3.985114	-2.018625	0.268595
31.	6.	0.	-2.778404	-2.876328	0.170490
32.	6.	0.	-1.862518	-2.865386	-1.076403
33.	6.	0.	-0.375595	-2.694917	-0.800748
34.	6.	0.	-6.656915	-0.383275	-0.399006
35.	6.	0.	-4.822657	-2.226647	1.509262
36.	8.	0.	-2.312279	-3.140742	-2.171153
37.	8.	0.	-2.435549	-3.659309	1.051579
38.	8.	0.	-0.974641	4.319247	1.021821
39.	6.	0.	0.513604	5.089577	-1.535724
40.	6.	0.	3.064339	3.167520	-1.505859
41.	1.	0.	-2.481988	1.851810	1.379377
42.	1.	0.	-3.185719	1.762064	-1.603199
43.	1.	0.	1.101536	2.495498	-1.995047
44.	1.	0.	-0.956273	2.194660	-1.278909
45.	1.	0.	1.492303	4.504087	0.279917
46.	1.	0.	-0.620840	-1.499639	1.018526
47.	1.	0.	1.013763	-1.560166	0.428281
48.	1.	0.	4.994263	-3.996845	-2.425807
49.	1.	0.	4.000616	-4.635555	-0.239681
50.	1.	0.	4.841440	0.177487	-1.389159
51.	1.	0.	5.408954	-1.623130	-2.984830
52.	1.	0.	3.058293	-3.466683	2.167492
53.	1.	0.	2.783355	-1.186144	3.167279
54.	1.	0.	4.379689	1.493956	0.694886
55.	1.	0.	4.054844	1.330341	2.402144
56.	1.	0.	1.497953	1.408188	3.208453
57.	1.	0.	2.534850	2.882029	1.372076
58.	1.	0.	-5.465649	2.083212	-0.352441
59.	1.	0.	-4.778699	1.146902	0.976250
60.	1.	0.	-5.270188	0.230025	-1.896230
61.	1.	0.	-3.454007	-1.081891	-1.505105
62.	1.	0.	0.132214	-2.635843	-1.765032
63.	1.	0.	-0.052531	-3.608799	-0.288588
64.	1.	0.	-7.350668	0.406573	-0.706017
65.	1.	0.	-6.753166	-0.502560	0.682422
66.	1.	0.	-6.979744	-1.315041	-0.873112
67.	1.	0.	-5.062225	-1.281012	2.001365
68.	1.	0.	-5.765071	-2.733308	1.275854
69.	1.	0.	-4.272522	-2.854205	2.212484
70.	1.	0.	-0.113173	4.669109	-2.330365
71.	1.	0.	1.410357	5.507480	-2.001134
72.	1.	0.	-0.037169	5.905351	-1.061198

73.	1.	0.	3.576271	2.298971	-1.932803
74.	1.	0.	3.659570	3.530967	-0.660391
75.	1.	0.	3.067745	3.952896	-2.266632

**1-c**			Standard Orientation (Ångstroms)		
Center number	Atom number	Type	X	Y	Z
1.	6.	0.	1.320608	2.769974	-1.194462
2.	6.	0.	1.403877	1.607396	-0.177722
3.	6.	0.	-0.014082	1.022008	0.195477
4.	6.	0.	-1.182693	2.023481	-0.153706
5.	6.	0.	-0.722162	3.439552	0.178254
6.	6.	0.	0.466809	3.940908	-0.648088
7.	6.	0.	-2.518239	1.666069	0.440922
8.	6.	0.	-0.247893	-0.294470	-0.591735
9.	6.	0.	0.360495	-1.584370	-0.053815
10.	6.	0.	5.168053	-2.762041	-1.658648
11.	6.	0.	4.636469	-3.174516	-0.443401
12.	6.	0.	4.182505	-2.182416	0.432939
13.	6.	0.	4.244264	-0.796270	0.115808
14.	6.	0.	4.798191	-0.413686	-1.120684
15.	6.	0.	5.251235	-1.394401	-1.992509
16.	7.	0.	3.625326	-2.285912	1.690543
17.	6.	0.	3.337334	-1.023520	2.167766
18.	6.	0.	3.691476	-0.072813	1.238583
19.	6.	0.	3.533223	1.417063	1.365200
20.	8.	0.	-0.829996	-0.264267	-1.659086
21.	6.	0.	0.063677	0.809404	1.717120
22.	7.	0.	1.178264	1.439235	2.172650
23.	6.	0.	2.094147	1.972558	1.178561
24.	1.	0.	1.995081	0.809751	-0.638811
25.	8.	0.	-0.743266	0.207994	2.414641
26.	6.	0.	-3.592625	1.390283	-0.302189
27.	6.	0.	-4.936301	0.980221	0.239279
28.	6.	0.	-5.570008	-0.207871	-0.540866
29.	6.	0.	-4.587857	-1.308523	-0.893567
30.	6.	0.	-3.726587	-1.982072	-0.104190
31.	6.	0.	-2.762513	-2.852688	-0.838764
32.	6.	0.	-1.469816	-3.315866	-0.145017
33.	6.	0.	-0.150230	-2.863316	-0.751517
34.	6.	0.	-6.831270	-0.739636	0.169067
35.	6.	0.	-3.571792	-1.836219	1.391501
36.	8.	0.	-1.525847	-4.067661	0.812016

37.	8.	0.	-2.901568	-3.248007	-1.988992
38.	8.	0.	-1.261617	4.143875	1.009431
39.	6.	0.	-0.073549	4.862752	-1.761028
40.	6.	0.	2.707367	3.236220	-1.660191
41.	1.	0.	-2.581560	1.628975	1.523927
42.	1.	0.	-3.507495	1.432685	-1.389315
43.	1.	0.	0.807127	2.360316	-2.075240
44.	1.	0.	-1.265472	1.961359	-1.246070
45.	1.	0.	1.068514	4.560062	0.029231
46.	1.	0.	0.207699	-1.642611	1.027024
47.	1.	0.	1.445678	-1.503505	-0.200052
48.	1.	0.	5.528969	-3.505746	-2.362324
49.	1.	0.	4.578587	-4.227362	-0.182933
50.	1.	0.	4.880418	0.635994	-1.388216
51.	1.	0.	5.678861	-1.107076	-2.948215
52.	1.	0.	3.450810	-3.145854	2.184824
53.	1.	0.	2.894920	-0.902149	3.146065
54.	1.	0.	4.182975	1.918487	0.640223
55.	1.	0.	3.882664	1.750732	2.351419
56.	1.	0.	1.361280	1.469685	3.166199
57.	1.	0.	2.158936	3.062487	1.290703
58.	1.	0.	-5.644413	1.819320	0.170018
59.	1.	0.	-4.846585	0.745621	1.305074
60.	1.	0.	-5.902119	0.205765	-1.502275
61.	1.	0.	-4.534960	-1.567004	-1.949739
62.	1.	0.	-0.264782	-2.685393	-1.822568
63.	1.	0.	0.572013	-3.667405	-0.585644
64.	1.	0.	-7.549614	0.071849	0.330000
65.	1.	0.	-6.591005	-1.174789	1.143647
66.	1.	0.	-7.322738	-1.513113	-0.428556
67.	1.	0.	-4.450904	-1.369190	1.835370
68.	1.	0.	-3.430423	-2.813008	1.859813
69.	1.	0.	-2.703127	-1.220058	1.654873
70.	1.	0.	-0.718605	4.310683	-2.453727
71.	1.	0.	0.742539	5.302721	-2.341140
72.	1.	0.	-0.658570	5.675752	-1.323830
73.	1.	0.	3.283948	2.402649	-2.074039
74.	1.	0.	3.285808	3.675750	-0.839625
75.	1.	0.	2.626096	3.995725	-2.442810

**1-d**			Standard Orientation (Ångstroms)		
Center number	Atom number	Type	X	Y	Z

1.	6.	0.	1.734654	2.808806	-1.167189
2.	6.	0.	1.796243	1.608252	-0.194911
3.	6.	0.	0.388566	1.080949	0.256299
4.	6.	0.	-0.789092	2.041228	-0.220457
5.	6.	0.	-0.363377	3.484919	0.102716
6.	6.	0.	0.921661	3.976767	-0.571366
7.	6.	0.	-2.163483	1.737491	0.317205
8.	6.	0.	0.125077	-0.318690	-0.370137
9.	6.	0.	-0.813856	-1.278973	0.333231
10.	6.	0.	4.796471	-3.419128	-1.657493
11.	6.	0.	4.038435	-3.700795	-0.528275
12.	6.	0.	3.726455	-2.634069	0.321171
13.	6.	0.	4.154051	-1.303180	0.063467
14.	6.	0.	4.921913	-1.053056	-1.088317
15.	6.	0.	5.234712	-2.107834	-1.934639
16.	7.	0.	3.008027	-2.608277	1.498743
17.	6.	0.	2.978446	-1.316612	1.988856
18.	6.	0.	3.663396	-0.475421	1.141238
19.	6.	0.	3.869458	1.007722	1.287950
20.	8.	0.	0.611316	-0.576134	-1.454956
21.	6.	0.	0.448921	1.075307	1.799008
22.	7.	0.	1.627349	1.638805	2.167717
23.	6.	0.	2.600558	1.883463	1.116349
24.	1.	0.	2.282543	0.795536	-0.736466
25.	8.	0.	-0.408543	0.671065	2.575647
26.	6.	0.	-3.256646	1.648749	-0.446812
27.	6.	0.	-4.653028	1.424266	0.076066
28.	6.	0.	-5.488978	0.413987	-0.759398
29.	6.	0.	-4.755023	-0.888765	-1.014457
30.	6.	0.	-4.261665	-1.795578	-0.146245
31.	6.	0.	-3.368224	-2.819517	-0.769522
32.	6.	0.	-2.170725	-3.348903	0.048604
33.	6.	0.	-0.847422	-2.689668	-0.286538
34.	6.	0.	-6.901786	0.226558	-0.172386
35.	6.	0.	-4.384635	-1.790568	1.359720
36.	8.	0.	-2.319315	-4.236897	0.866050
37.	8.	0.	-3.453325	-3.213901	-1.923205
38.	8.	0.	-1.013365	4.206994	0.831085
39.	6.	0.	0.547429	5.063874	-1.599141
40.	6.	0.	3.133375	3.232693	-1.638548
41.	1.	0.	-2.243109	1.633054	1.395240
42.	1.	0.	-3.163510	1.771572	-1.528406
43.	1.	0.	1.201827	2.445619	-2.056519
44.	1.	0.	-0.806278	1.960347	-1.316222

45.	1.	0.	1.502131	4.463257	0.224639
46.	1.	0.	-1.797573	-0.794861	0.268894
47.	1.	0.	-0.593157	-1.306528	1.402074
48.	1.	0.	5.056224	-4.223330	-2.339098
49.	1.	0.	3.700552	-4.710704	-0.314581
50.	1.	0.	5.271332	-0.049152	-1.314156
51.	1.	0.	5.826467	-1.924040	-2.826277
52.	1.	0.	2.596794	-3.408553	1.950931
53.	1.	0.	2.467081	-1.100904	2.915977
54.	1.	0.	4.605361	1.344681	0.549501
55.	1.	0.	4.306590	1.243761	2.267970
56.	1.	0.	1.846408	1.719861	3.151607
57.	1.	0.	2.921814	2.931172	1.165228
58.	1.	0.	-5.201197	2.378090	0.064476
59.	1.	0.	-4.606005	1.113227	1.125185
60.	1.	0.	-5.622039	0.875764	-1.746711
61.	1.	0.	-4.539135	-1.103454	-2.059770
62.	1.	0.	-0.744543	-2.609548	-1.373332
63.	1.	0.	-0.030565	-3.305840	0.098279
64.	1.	0.	-7.419136	1.190427	-0.117439
65.	1.	0.	-6.876205	-0.195279	0.836204
66.	1.	0.	-7.499484	-0.443084	-0.797516
67.	1.	0.	-4.681598	-2.778879	1.724079
68.	1.	0.	-3.432662	-1.551374	1.847993
69.	1.	0.	-5.120422	-1.064544	1.703216
70.	1.	0.	-0.023773	4.639575	-2.432900
71.	1.	0.	1.439577	5.541476	-2.012535
72.	1.	0.	-0.063867	5.833125	-1.121435
73.	1.	0.	3.679284	2.376240	-2.047505
74.	1.	0.	3.730929	3.655987	-0.822991
75.	1.	0.	3.076315	3.988948	-2.426115

**1-e**			Standard Orientation (Ångstroms)		
Center number	Atom number	Type	X	Y	Z
1.	6.	0.	0.821495	3.228786	-1.213833
2.	6.	0.	1.222005	1.903459	-0.522997
3.	6.	0.	-0.021348	1.101381	0.030142
4.	6.	0.	-1.305056	2.008168	0.151416
5.	6.	0.	-0.875397	3.372369	0.682954
6.	6.	0.	0.052639	4.161848	-0.245769
7.	6.	0.	-2.448562	1.394828	0.913338
8.	6.	0.	-0.335981	-0.072385	-0.935409

9.	6.	0.	0.432468	-1.377354	-0.763730
10.	6.	0.	4.534743	-3.578624	1.299756
11.	6.	0.	4.599953	-3.261529	-0.050506
12.	6.	0.	4.395610	-1.924741	-0.409535
13.	6.	0.	4.120138	-0.908106	0.546294
14.	6.	0.	4.071690	-1.264927	1.906379
15.	6.	0.	4.277903	-2.588138	2.269784
16.	7.	0.	4.411981	-1.324190	-1.652737
17.	6.	0.	4.168345	0.026961	-1.506742
18.	6.	0.	3.970742	0.333928	-0.179803
19.	6.	0.	3.690667	1.702199	0.374423
20.	8.	0.	-1.116038	0.094854	-1.853449
21.	6.	0.	0.434951	0.616159	1.417328
22.	7.	0.	1.625264	1.205400	1.700843
23.	6.	0.	2.208260	2.058940	0.679968
24.	1.	0.	1.724701	1.279542	-1.269632
25.	8.	0.	-0.166203	-0.155899	2.153088
26.	6.	0.	-3.649800	1.187528	0.368496
27.	6.	0.	-4.813273	0.534554	1.064614
28.	6.	0.	-5.524300	-0.536065	0.185043
29.	6.	0.	-4.570601	-1.419612	-0.596302
30.	6.	0.	-3.517369	-2.143794	-0.167272
31.	6.	0.	-2.679426	-2.729287	-1.255035
32.	6.	0.	-1.263317	-3.240232	-0.938627
33.	6.	0.	-0.095501	-2.546292	-1.623144
34.	6.	0.	-6.555088	-1.341255	1.000981
35.	6.	0.	-3.053490	-2.302611	1.261526
36.	8.	0.	-1.119678	-4.212698	-0.219849
37.	8.	0.	-3.024318	-2.855486	-2.422903
38.	8.	0.	-1.249095	3.828201	1.746017
39.	6.	0.	-0.789558	5.229175	-0.974337
40.	6.	0.	2.020102	3.936438	-1.862940
41.	1.	0.	-2.259180	1.112200	1.943887
42.	1.	0.	-3.815198	1.478727	-0.670345
43.	1.	0.	0.136900	2.947143	-2.025495
44.	1.	0.	-1.625614	2.154480	-0.887797
45.	1.	0.	0.759909	4.688944	0.407035
46.	1.	0.	0.460039	-1.651491	0.294492
47.	1.	0.	1.475159	-1.164654	-1.035462
48.	1.	0.	4.685042	-4.606700	1.614116
49.	1.	0.	4.802971	-4.022450	-0.798473
50.	1.	0.	3.881179	-0.516264	2.669353
51.	1.	0.	4.237783	-2.869440	3.317444
52.	1.	0.	4.617214	-1.787607	-2.522706

53.	1.	0.	4.172484	0.680094	-2.368542
54.	1.	0.	4.066281	2.459907	-0.321793
55.	1.	0.	4.259392	1.846801	1.302893
56.	1.	0.	2.052774	1.047159	2.602441
57.	1.	0.	2.210406	3.098678	1.034215
58.	1.	0.	-5.571450	1.287885	1.324968
59.	1.	0.	-4.476298	0.101350	2.011819
60.	1.	0.	-6.090628	0.022902	-0.571597
61.	1.	0.	-4.727379	-1.440096	-1.673430
62.	1.	0.	-0.402444	-2.180945	-2.605889
63.	1.	0.	0.696815	-3.291824	-1.731561
64.	1.	0.	-7.272427	-0.666207	1.480666
65.	1.	0.	-6.073890	-1.933528	1.784732
66.	1.	0.	-7.113951	-2.029182	0.359636
67.	1.	0.	-3.835429	-2.019118	1.965723
68.	1.	0.	-2.772609	-3.339387	1.461311
69.	1.	0.	-2.176630	-1.679849	1.478235
70.	1.	0.	-1.539726	4.765285	-1.624506
71.	1.	0.	-0.161630	5.875534	-1.593996
72.	1.	0.	-1.309617	5.856357	-0.245991
73.	1.	0.	2.543973	3.270856	-2.557360
74.	1.	0.	2.742219	4.281029	-1.114608
75.	1.	0.	1.698930	4.813012	-2.432509

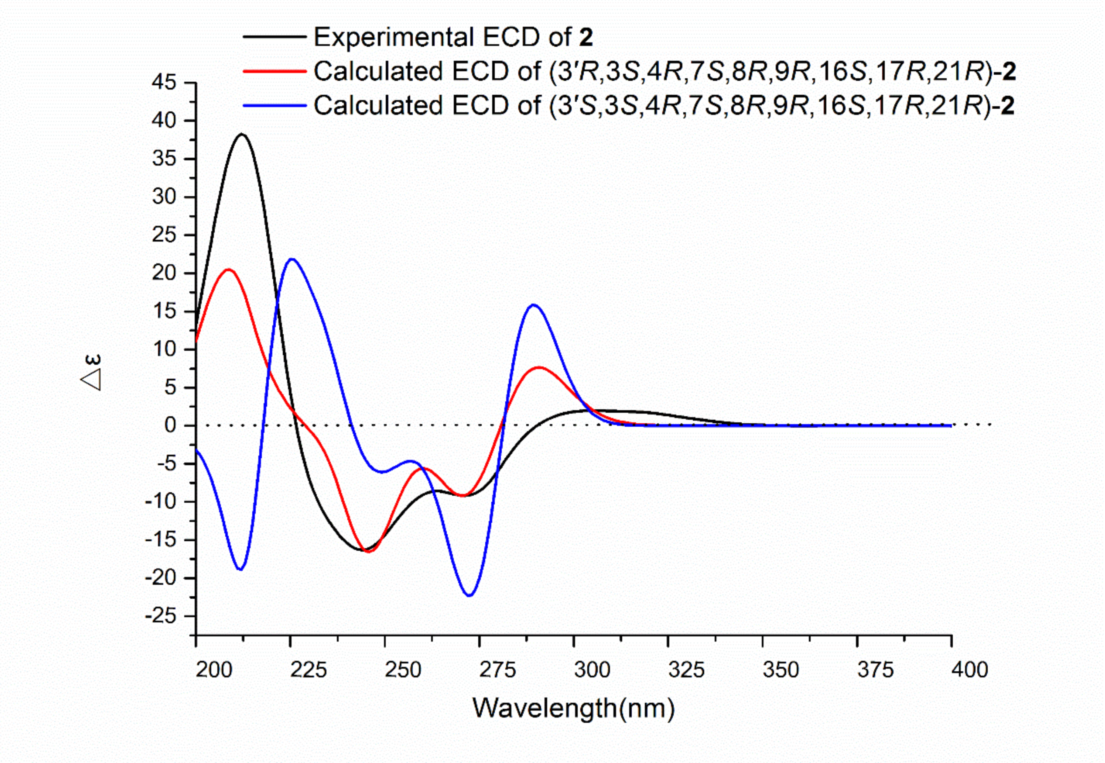

Figure S22. Experimental ECD spectra of **2** and calculated ECD spectra for (3'*R*, 3*S*, 4*R*, 7*S*, 8*R*, 9*R*, 16*S*, 17*R*, 21*R*)-**2** and (3'*S*, 3*S*, 4*R*, 7*S*, 8*R*, 9*R*, 16*S*, 17*R*, 21*R*)-**2**

Table S5. Gibbs free energies[a] and equilibrium populations[b] of low-energy conformers of (3'*R*, 3*S*, 4*R*, 7*S*, 8*R*, 9*R*, 16*S*, 17*R*, 21*R*)-**2**.

Conformers	In MeOH	
	Δ*G*	*P* (%)
**2-a**	0.00	49.0%
**2-b**	0.53	20.2%
**2-c**	0.87	11.3%
**2-d**	1.18	6.6%
**2-e**	1.49	3.9%

[a]B3LYP/6-31+G (d, p), in kcal/mol, [b]From Δ*G* values at 298.15K.

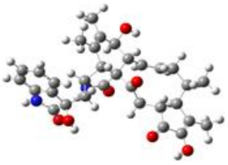

2-a 49.0%

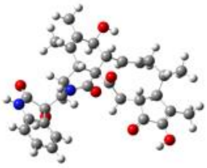

2-b 20.2%

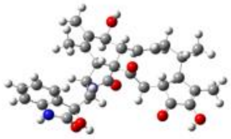

2-c 11.3%

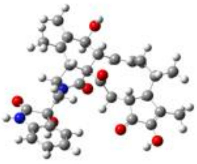

2-d 6.6%

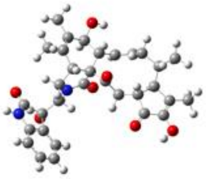

2-e 3.9%

Figure S23. Structures and populations of the low-energy conformers of (3'*R*, 3*S*, 4*R*, 7*S*, 8*R*, 9*R*, 16*S*, 17*R*, 21*R*)-**2**

Table S6. Cartesian coordinates for the low-energy reoptimized MMFF conformers of (3'*R*, 3*S*, 4*R*, 7*S*, 8*R*, 9*R*, 16*S*, 17*R*, 21*R*)-**2** at B3LYP/6-311+G (d, p) level of theory in CH_3OH.

**2-a**			Standard Orientation (Ångstroms)		
Center number	Atomic number	Atomic Type	X	Y	Z
1.	6.	0.	1.817599	2.496850	-0.612802
2.	6.	0.	1.182775	1.118079	-0.539443
3.	6.	0.	-0.186874	1.042777	0.181074
4.	6.	0.	-0.972297	2.392873	0.128443
5.	6.	0.	-0.084463	3.557791	0.606403
6.	6.	0.	1.264855	3.599188	-0.078867
7.	6.	0.	-2.270547	2.420350	0.913308

8.	6.	0.	-1.104657	0.013315	-0.538272
9.	6.	0.	-2.074498	-0.837583	0.264090
10.	6.	0.	7.253911	-0.520305	-1.153960
11.	6.	0.	6.903944	-0.709378	0.189500
12.	6.	0.	5.642171	-1.224349	0.454964
13.	6.	0.	4.736056	-1.539457	-0.568053
14.	6.	0.	5.096988	-1.352821	-1.893370
15.	6.	0.	6.368138	-0.837481	-2.185048
16.	7.	0.	5.053016	-1.509915	1.701624
17.	6.	0.	3.785260	-2.018967	1.567057
18.	6.	0.	3.462623	-2.085185	0.044275
19.	6.	0.	2.183874	-1.298916	-0.318284
20.	8.	0.	-1.055046	-0.069095	-1.753300
21.	6.	0.	0.169559	0.598536	1.613062
22.	7.	0.	1.452876	0.144964	1.592689
23.	6.	0.	2.088662	0.126719	0.277555
24.	1.	0.	1.069108	0.743359	-1.561001
25.	8.	0.	-0.562253	0.626424	2.594479
26.	6.	0.	-3.516133	2.241186	0.452781
27.	6.	0.	-3.947631	1.817574	-0.928953
28.	6.	0.	-5.074329	0.737850	-0.926960
29.	6.	0.	-4.693003	-0.502028	-0.061174
30.	6.	0.	-3.358817	-1.187857	-0.510410
31.	6.	0.	-6.408214	1.392397	-0.521662
32.	6.	0.	3.151157	2.528611	-1.342087
33.	6.	0.	1.928201	4.954361	-0.082386
34.	8.	0.	-0.741843	4.797657	0.334385
35.	6.	0.	-3.663185	-2.674954	-0.463156
36.	6.	0.	-5.097945	-2.834671	-0.263389
37.	6.	0.	-5.705342	-1.654207	-0.014086
38.	8.	0.	-2.877190	-3.612631	-0.541024
39.	8.	0.	-5.641275	-4.073220	-0.241032
40.	6.	0.	-7.144290	-1.561908	0.396679
41.	1.	0.	-3.169526	-0.967142	-1.570203
42.	1.	0.	-4.566514	-0.139652	0.970643
43.	8.	0.	3.040936	-2.370291	2.469269
44.	8.	0.	3.322364	-3.454306	-0.326277
45.	1.	0.	-1.175349	2.583706	-0.930815
46.	1.	0.	0.068145	3.457993	1.696700
47.	1.	0.	-2.152674	2.635334	1.973679
48.	1.	0.	-1.525960	-1.774848	0.452019
49.	1.	0.	-2.269515	-0.391088	1.238929
50.	1.	0.	8.235140	-0.121120	-1.392286
51.	1.	0.	7.597464	-0.465692	0.988202

52.	1.	0.	4.410105	-1.612852	-2.693228
53.	1.	0.	6.666038	-0.687982	-3.217733
54.	1.	0.	5.510185	-1.426021	2.598501
55.	1.	0.	1.322832	-1.885621	0.021899
56.	1.	0.	2.131630	-1.261163	-1.410996
57.	1.	0.	1.804715	-0.395085	2.374253
58.	1.	0.	3.098612	0.542563	0.356614
59.	1.	0.	-4.322874	2.375369	1.173191
60.	1.	0.	-3.094011	1.467179	-1.512284
61.	1.	0.	-4.343501	2.688307	-1.471471
62.	1.	0.	-5.177492	0.394026	-1.965688
63.	1.	0.	-6.552673	2.324883	-1.077182
64.	1.	0.	-6.439154	1.639016	0.545679
65.	1.	0.	-7.265827	0.754775	-0.736138
66.	1.	0.	3.312103	1.607388	-1.909931
67.	1.	0.	3.204617	3.359320	-2.051171
68.	1.	0.	4.002545	2.633224	-0.658025
69.	1.	0.	1.932778	5.375992	0.929814
70.	1.	0.	1.367866	5.661032	-0.702309
71.	1.	0.	2.959054	4.914251	-0.436005
72.	1.	0.	-1.633515	4.729947	0.704594
73.	1.	0.	-4.889880	-4.683348	-0.356296
74.	1.	0.	-7.481255	-2.530434	0.776861
75.	1.	0.	-7.298059	-0.806666	1.173403
76.	1.	0.	-7.799371	-1.301840	-0.443561
77.	1.	0.	2.636138	-3.829103	0.248202

**2-b**			Standard Orientation (Ångstroms)		
Center number	Atom number	Type	X	Y	Z
1.	6.	0.	1.204672	3.250158	-0.838158
2.	6.	0.	0.964533	1.772734	-0.561581
3.	6.	0.	-0.368285	1.406977	0.147592
4.	6.	0.	-1.461739	2.507831	-0.029763
5.	6.	0.	-0.898112	3.895431	0.334618
6.	6.	0.	0.386913	4.225312	-0.401405
7.	6.	0.	-2.742174	2.279642	0.751167
8.	6.	0.	-0.976675	0.129339	-0.494177
9.	6.	0.	-1.722861	-0.876691	0.367394
10.	6.	0.	5.762884	-4.130104	-0.942727
11.	6.	0.	6.360675	-2.865509	-1.025947
12.	6.	0.	5.675117	-1.794976	-0.466509
13.	6.	0.	4.427468	-1.956808	0.154161

14.	6.	0.	3.847779	-3.214464	0.233251
15.	6.	0.	4.526182	-4.309456	-0.320049
16.	7.	0.	6.054627	-0.443449	-0.399379
17.	6.	0.	5.137972	0.327716	0.285678
18.	6.	0.	3.930854	-0.608608	0.641521
19.	6.	0.	2.663307	-0.142855	-0.104471
20.	8.	0.	-0.874880	-0.033663	-1.698051
21.	6.	0.	0.029776	1.179705	1.620948
22.	7.	0.	1.379049	1.072115	1.660213
23.	6.	0.	2.076746	1.196942	0.388821
24.	1.	0.	1.000384	1.246127	-1.520295
25.	8.	0.	-0.728001	1.090513	2.581505
26.	6.	0.	-3.894228	1.757823	0.307876
27.	6.	0.	-4.174527	1.125368	-1.032561
28.	6.	0.	-4.998810	-0.195434	-0.941972
29.	6.	0.	-4.333428	-1.234155	0.012456
30.	6.	0.	-2.860970	-1.593292	-0.383198
31.	6.	0.	-6.458539	0.135346	-0.578522
32.	6.	0.	2.448076	3.492651	-1.666104
33.	6.	0.	0.637645	5.705548	-0.563777
34.	8.	0.	-1.857120	4.898293	-0.010653
35.	6.	0.	-2.784929	-3.101821	-0.226469
36.	6.	0.	-4.137209	-3.601848	-0.014974
37.	6.	0.	-5.025494	-2.597098	0.145243
38.	8.	0.	-1.788607	-3.817082	-0.234835
39.	8.	0.	-4.352425	-4.932795	0.099878
40.	6.	0.	-6.449401	-2.839345	0.546676
41.	1.	0.	-2.710698	-1.407394	-1.455895
42.	1.	0.	-4.322102	-0.778544	1.014654
43.	8.	0.	5.257708	1.509622	0.548833
44.	8.	0.	3.638068	-0.571110	2.036661
45.	1.	0.	-1.683193	2.546971	-1.101673
46.	1.	0.	-0.715803	3.926715	1.424558
47.	1.	0.	-2.703153	2.605265	1.788813
48.	1.	0.	-0.961290	-1.629380	0.627767
49.	1.	0.	-2.045629	-0.424607	1.304936
50.	1.	0.	6.276946	-4.985094	-1.371398
51.	1.	0.	7.324224	-2.732780	-1.508074
52.	1.	0.	2.885957	-3.347266	0.719998
53.	1.	0.	4.086424	-5.299955	-0.266702
54.	1.	0.	6.933935	-0.066221	-0.722434
55.	1.	0.	1.907970	-0.932757	-0.019815
56.	1.	0.	2.913549	-0.057853	-1.167821
57.	1.	0.	1.865973	0.787520	2.498498

58.	1.	0.	2.895069	1.909998	0.501912
59.	1.	0.	-4.725220	1.748553	1.013030
60.	1.	0.	-3.246061	0.947797	-1.578301
61.	1.	0.	-4.758557	1.821881	-1.651520
62.	1.	0.	-5.000162	-0.630911	-1.951047
63.	1.	0.	-6.817455	0.969185	-1.190847
64.	1.	0.	-6.565230	0.430543	0.471375
65.	1.	0.	-7.131986	-0.703900	-0.753687
66.	1.	0.	2.389684	2.911950	-2.596433
67.	1.	0.	2.579753	4.537679	-1.944384
68.	1.	0.	3.362306	3.170035	-1.153092
69.	1.	0.	0.512992	6.213218	0.399805
70.	1.	0.	-0.096352	6.154693	-1.239413
71.	1.	0.	1.639086	5.929106	-0.931223
72.	1.	0.	-2.700991	4.625249	0.376459
73.	1.	0.	-3.469305	-5.339258	0.028730
74.	1.	0.	-6.543359	-3.835779	0.987487
75.	1.	0.	-6.797770	-2.099156	1.273432
76.	1.	0.	-7.133715	-2.800664	-0.309405
77.	1.	0.	4.400769	-0.918226	2.519234

**2-c**			Standard Orientation (Ångstroms)		
Center number	Atom number	Type	X	Y	Z
1.	6.	0.	1.835804	2.504870	-0.607365
2.	6.	0.	1.181375	1.135562	-0.542542
3.	6.	0.	-0.189323	1.078202	0.179003
4.	6.	0.	-0.963538	2.437352	0.121931
5.	6.	0.	-0.058674	3.576690	0.630008
6.	6.	0.	1.299972	3.604860	-0.049985
7.	6.	0.	-2.284334	2.482829	0.867117
8.	6.	0.	-1.105342	0.040678	-0.529488
9.	6.	0.	-2.066672	-0.807594	0.283667
10.	6.	0.	7.218262	-0.577295	-1.174503
11.	6.	0.	6.870921	-0.761389	0.170338
12.	6.	0.	5.604584	-1.262575	0.440776
13.	6.	0.	4.691549	-1.568680	-0.578952
14.	6.	0.	5.049964	-1.387206	-1.905664
15.	6.	0.	6.325557	-0.885815	-2.202257
16.	7.	0.	5.016822	-1.540463	1.689655
17.	6.	0.	3.743035	-2.036379	1.559886
18.	6.	0.	3.414221	-2.099513	0.038293
19.	6.	0.	2.143495	-1.297665	-0.318160

20.	8.	0.	-1.053525	-0.053674	-1.744004
21.	6.	0.	0.166320	0.642987	1.614112
22.	7.	0.	1.442424	0.166198	1.590887
23.	6.	0.	2.073268	0.131181	0.274010
24.	1.	0.	1.063308	0.764485	-1.564772
25.	8.	0.	-0.559031	0.695578	2.597953
26.	6.	0.	-3.514121	2.256727	0.389462
27.	6.	0.	-3.924550	1.789483	-0.985683
28.	6.	0.	-5.053051	0.711900	-0.974125
29.	6.	0.	-4.682336	-0.506573	-0.075743
30.	6.	0.	-3.339077	-1.194698	-0.490085
31.	6.	0.	-6.390959	1.375805	-0.598113
32.	6.	0.	3.168076	2.526268	-1.338617
33.	6.	0.	1.994829	4.945490	-0.016665
34.	8.	0.	-0.734970	4.830183	0.505359
35.	6.	0.	-3.631844	-2.681915	-0.401253
36.	6.	0.	-5.068524	-2.846472	-0.221220
37.	6.	0.	-5.688169	-1.663509	-0.016942
38.	8.	0.	-2.837023	-3.614977	-0.439191
39.	8.	0.	-5.604143	-4.088100	-0.173812
40.	6.	0.	-7.135521	-1.568326	0.361600
41.	1.	0.	-3.142883	-1.003964	-1.554489
42.	1.	0.	-4.572354	-0.119819	0.949003
43.	8.	0.	2.998154	-2.378896	2.464703
44.	8.	0.	3.255771	-3.466357	-0.332161
45.	1.	0.	-1.137582	2.620449	-0.947471
46.	1.	0.	0.076159	3.461814	1.714468
47.	1.	0.	-2.198627	2.779394	1.908352
48.	1.	0.	-1.505278	-1.731761	0.497732
49.	1.	0.	-2.275161	-0.340251	1.245978
50.	1.	0.	8.203006	-0.189149	-1.416570
51.	1.	0.	7.569939	-0.524701	0.966350
52.	1.	0.	4.357575	-1.640845	-2.702833
53.	1.	0.	6.621650	-0.740856	-3.236125
54.	1.	0.	5.478397	-1.461787	2.584762
55.	1.	0.	1.277533	-1.871655	0.030824
56.	1.	0.	2.083632	-1.263229	-1.410596
57.	1.	0.	1.789335	-0.373353	2.374854
58.	1.	0.	3.090409	0.530190	0.347829
59.	1.	0.	-4.335149	2.417800	1.087434
60.	1.	0.	-3.065244	1.422433	-1.550322
61.	1.	0.	-4.313989	2.644814	-1.557790
62.	1.	0.	-5.145058	0.344141	-2.005898
63.	1.	0.	-6.526972	2.297511	-1.173406

64.	1.	0.	-6.433374	1.643641	0.463510
65.	1.	0.	-7.247279	0.735342	-0.810243
66.	1.	0.	3.299048	1.625070	-1.945175
67.	1.	0.	3.247930	3.385100	-2.010579
68.	1.	0.	4.022852	2.573750	-0.652302
69.	1.	0.	1.956717	5.363507	0.995593
70.	1.	0.	1.497948	5.679605	-0.660677
71.	1.	0.	3.041072	4.879650	-0.317833
72.	1.	0.	-0.987103	4.933522	-0.423280
73.	1.	0.	-4.846640	-4.695624	-0.257964
74.	1.	0.	-7.478482	-2.530673	0.751842
75.	1.	0.	-7.306855	-0.799455	1.121139
76.	1.	0.	-7.772749	-1.323908	-0.496866
77.	1.	0.	2.566485	-3.833071	0.243900

**2-d**			Standard Orientation (Ångstroms)		
Center number	Atom number	Type	X	Y	Z
1.	6.	0.	1.094230	3.626958	-0.805876
2.	6.	0.	0.926535	2.125191	-0.654371
3.	6.	0.	-0.370898	1.675899	0.065963
4.	6.	0.	-1.568408	2.654745	-0.154141
5.	6.	0.	-1.146201	4.084734	0.231201
6.	6.	0.	0.177251	4.515573	-0.381629
7.	6.	0.	-2.851133	2.296958	0.578260
8.	6.	0.	-0.833755	0.314093	-0.507265
9.	6.	0.	-1.419066	-0.731141	0.423484
10.	6.	0.	5.075728	-4.203003	-1.206482
11.	6.	0.	5.867409	-3.074885	-0.952012
12.	6.	0.	5.250325	-1.981273	-0.358774
13.	6.	0.	3.886920	-1.990967	-0.026971
14.	6.	0.	3.110699	-3.112676	-0.284827
15.	6.	0.	3.718746	-4.227943	-0.879042
16.	7.	0.	5.809573	-0.744354	0.008309
17.	6.	0.	4.892036	0.087430	0.618471
18.	6.	0.	3.515933	-0.657172	0.594187
19.	6.	0.	2.485038	0.106431	-0.258681
20.	8.	0.	-0.742703	0.114603	-1.707591
21.	6.	0.	0.060998	1.549435	1.542211
22.	7.	0.	1.417439	1.489649	1.553614
23.	6.	0.	2.055343	1.503158	0.243503
24.	1.	0.	0.962091	1.671693	-1.650348
25.	8.	0.	-0.674953	1.492549	2.519730

26.	6.	0.	-3.884283	1.552404	0.162752
27.	6.	0.	-4.054371	0.779012	-1.122695
28.	6.	0.	-4.703432	-0.630276	-0.945443
29.	6.	0.	-3.919184	-1.520143	0.067430
30.	6.	0.	-2.402509	-1.676070	-0.280463
31.	6.	0.	-6.193990	-0.467537	-0.592873
32.	6.	0.	2.394467	4.039773	-1.470748
33.	6.	0.	0.377158	6.013134	-0.427645
34.	8.	0.	-2.195417	5.003673	-0.088179
35.	6.	0.	-2.095478	-3.136385	-0.013846
36.	6.	0.	-3.361748	-3.827152	0.191770
37.	6.	0.	-4.400789	-2.964685	0.255020
38.	8.	0.	-0.998501	-3.685806	0.049308
39.	8.	0.	-3.377796	-5.167286	0.382940
40.	6.	0.	-5.789499	-3.402459	0.608866
41.	1.	0.	-2.261486	-1.545236	-1.362747
42.	1.	0.	-3.998286	-1.022936	1.046781
43.	8.	0.	5.122265	1.186491	1.086124
44.	8.	0.	2.981363	-0.777017	1.912526
45.	1.	0.	-1.746795	2.649911	-1.238638
46.	1.	0.	-1.073782	4.143965	1.325866
47.	1.	0.	-2.931000	2.743895	1.564924
48.	1.	0.	-0.552631	-1.316259	0.771328
49.	1.	0.	-1.840455	-0.257163	1.310257
50.	1.	0.	5.532465	-5.073103	-1.668611
51.	1.	0.	6.922106	-3.061536	-1.208917
52.	1.	0.	2.052424	-3.130934	-0.036621
53.	1.	0.	3.128601	-5.114240	-1.087586
54.	1.	0.	6.784847	-0.496033	-0.074848
55.	1.	0.	1.612922	-0.546881	-0.356767
56.	1.	0.	2.899913	0.217770	-1.267340
57.	1.	0.	1.914277	1.168348	2.372940
58.	1.	0.	2.938114	2.144176	0.282053
59.	1.	0.	-4.727303	1.490611	0.850629
60.	1.	0.	-3.107921	0.687723	-1.657733
61.	1.	0.	-4.720973	1.342887	-1.792517
62.	1.	0.	-4.646353	-1.121661	-1.927151
63.	1.	0.	-6.658051	0.271271	-1.254461
64.	1.	0.	-6.337051	-0.119880	0.436435
65.	1.	0.	-6.753494	-1.396344	-0.706765
66.	1.	0.	2.767086	3.238972	-2.118631
67.	1.	0.	2.274326	4.929930	-2.091967
68.	1.	0.	3.186729	4.253807	-0.742386
69.	1.	0.	0.102487	6.455538	0.536497

70.	1.	0.	-0.271427	6.491244	-1.170193
71.	1.	0.	1.409263	6.291007	-0.643271
72.	1.	0.	-2.360820	4.933669	-1.039220
73.	1.	0.	-2.442090	-5.438291	0.363928
74.	1.	0.	-6.270407	-2.702352	1.298912
75.	1.	0.	-6.434672	-3.480825	-0.274584
76.	1.	0.	-5.759095	-4.391208	1.074767
77.	1.	0.	3.538853	-1.393229	2.407650

**2-e**			Standard Orientation (Ångstroms)		
Center number	Atom number	Type	X	Y	Z
1.	6.	0.	1.225074	3.181509	-0.856145
2.	6.	0.	0.975950	1.710275	-0.555374
3.	6.	0.	-0.358365	1.359947	0.160884
4.	6.	0.	-1.429675	2.488568	0.015019
5.	6.	0.	-0.827294	3.859767	0.381196
6.	6.	0.	0.437150	4.171149	-0.397957
7.	6.	0.	-2.701007	2.278672	0.815101
8.	6.	0.	-1.004143	0.110680	-0.504518
9.	6.	0.	-1.767561	-0.898412	0.337589
10.	6.	0.	5.684426	-4.228861	-0.788104
11.	6.	0.	6.242932	-2.979984	-1.090913
12.	6.	0.	5.631857	-1.859142	-0.546757
13.	6.	0.	4.492640	-1.950980	0.266545
14.	6.	0.	3.945944	-3.192130	0.553089
15.	6.	0.	4.554981	-4.339047	0.024589
16.	7.	0.	6.000889	-0.503251	-0.681086
17.	6.	0.	5.205976	0.311309	0.082359
18.	6.	0.	4.040711	-0.552810	0.635452
19.	6.	0.	2.711496	-0.185159	-0.077176
20.	8.	0.	-0.916074	-0.022759	-1.712720
21.	6.	0.	0.043082	1.094798	1.625643
22.	7.	0.	1.396054	1.005311	1.667269
23.	6.	0.	2.093883	1.146122	0.396461
24.	1.	0.	1.006358	1.169009	-1.505697
25.	8.	0.	-0.711079	0.965164	2.583437
26.	6.	0.	-3.872294	1.794773	0.379142
27.	6.	0.	-4.186453	1.200617	-0.971304
28.	6.	0.	-5.045374	-0.098572	-0.900809
29.	6.	0.	-4.391177	-1.179976	0.012832
30.	6.	0.	-2.934477	-1.563106	-0.417343
31.	6.	0.	-6.489106	0.260864	-0.502058

32.	6.	0.	2.443308	3.397384	-1.727135
33.	6.	0.	0.702038	5.646587	-0.579046
34.	8.	0.	-1.776087	4.886274	0.083785
35.	6.	0.	-2.894275	-3.077477	-0.306634
36.	6.	0.	-4.255475	-3.549823	-0.087858
37.	6.	0.	-5.115160	-2.528558	0.117237
38.	8.	0.	-1.916620	-3.816198	-0.352182
39.	8.	0.	-4.502810	-4.877653	-0.009313
40.	6.	0.	-6.538511	-2.746028	0.534228
41.	1.	0.	-2.796216	-1.348899	-1.486247
42.	1.	0.	-4.352462	-0.754209	1.027409
43.	8.	0.	5.382601	1.493713	0.333279
44.	8.	0.	3.951303	-0.338983	2.046111
45.	1.	0.	-1.668853	2.544288	-1.052139
46.	1.	0.	-0.606107	3.871395	1.464514
47.	1.	0.	-2.636893	2.581551	1.858470
48.	1.	0.	-1.023435	-1.678695	0.565788
49.	1.	0.	-2.065914	-0.465349	1.291903
50.	1.	0.	6.142697	-5.124687	-1.195909
51.	1.	0.	7.122190	-2.897929	-1.722224
52.	1.	0.	3.066747	-3.275507	1.184790
53.	1.	0.	4.143419	-5.318189	0.246576
54.	1.	0.	6.862623	-0.174501	-1.093340
55.	1.	0.	1.994781	-0.997939	0.087228
56.	1.	0.	2.898341	-0.146641	-1.156875
57.	1.	0.	1.870402	0.659724	2.490278
58.	1.	0.	2.894762	1.882046	0.505672
59.	1.	0.	-4.693067	1.790491	1.096105
60.	1.	0.	-3.269436	1.009586	-1.531854
61.	1.	0.	-4.757675	1.927297	-1.567037
62.	1.	0.	-5.075916	-0.506309	-1.920956
63.	1.	0.	-6.835081	1.122474	-1.082532
64.	1.	0.	-6.570018	0.526115	0.558016
65.	1.	0.	-7.188158	-0.554153	-0.690533
66.	1.	0.	2.331560	2.832673	-2.662326
67.	1.	0.	2.599224	4.441713	-1.995253
68.	1.	0.	3.365260	3.038155	-1.253433
69.	1.	0.	0.624049	6.161381	0.385646
70.	1.	0.	-0.052827	6.102284	-1.226570
71.	1.	0.	1.690252	5.853597	-0.989405
72.	1.	0.	-2.614728	4.620328	0.486924
73.	1.	0.	-3.631877	-5.304341	-0.107328
74.	1.	0.	-6.652071	-3.752470	0.946707
75.	1.	0.	-6.856075	-2.019088	1.287975

76.	1.	0.	-7.234362	-2.663696	-0.309400
77.	1.	0.	4.373434	0.519891	2.213182

Table S7. Gibbs free energies[a] and equilibrium populations[b] of low-energy conformers of (3'*S*, 3*S*, 4*R*, 7*S*, 8*R*, 9*R*, 16*S*, 17*R*, 21*R*)-**2**

Conformers	In MeOH	
	Δ*G*	*P* (%)
**2-a**	0.00	37.0%
**2-b**	0.35	20.6%
**2-c**	0.46	16.9%
**2-d**	0.77	10.0%
**2-e**	1.14	5.4%

[a]B3LYP/6-31+G (d, p), in kcal/mol. [b]From Δ*G* values at 298.15K.

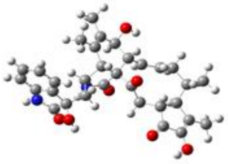

2-a 49.0%

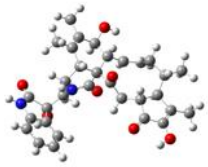

2-b 20.2%

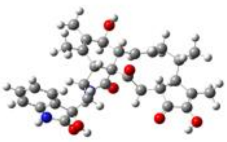

2-c 11.3%

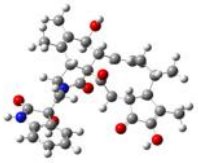

2-d 6.6%

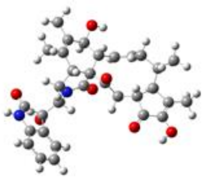

2-e 3.9%

Figure S24. Structures and populations of the low-energy conformers of (3'*S*, 3*S*, 4*R*, 7*S*, 8*R*, 9*R*, 16*S*, 17*R*, 21*R*)-**2**

Table S8. Cartesian coordinates for the low-energy reoptimized MMFF conformers of (3'*S*, 3*S*, 4*R*, 7*S*, 8*R*, 9*R*, 16*S*, 17*R*, 21*R*)-**2** at B3LYP/6-311+G (d, p) level of theory in CH_3OH.

**2-a**			Standard Orientation (Ångstroms)		
Center number	Atomic number	Atomic Type	X	Y	Z
1.	6.	0.	0.949807	3.583577	-0.841825
2.	6.	0.	0.870481	2.077076	-0.657461
3.	6.	0.	-0.385712	1.548397	0.082221
4.	6.	0.	-1.611187	2.508013	-0.052932
5.	6.	0.	-1.222577	3.949017	0.329478
6.	6.	0.	0.024151	4.439295	-0.375677
7.	6.	0.	-2.838558	2.113057	0.747186

8.	6.	0.	-0.850879	0.208896	-0.550368
9.	6.	0.	-1.452001	-0.877500	0.327637
10.	6.	0.	5.604598	-3.816379	-1.277849
11.	6.	0.	5.151609	-3.684856	0.041502
12.	6.	0.	4.686719	-2.437685	0.436590
13.	6.	0.	4.653632	-1.344739	-0.440558
14.	6.	0.	5.108570	-1.486781	-1.742712
15.	6.	0.	5.591273	-2.734904	-2.160596
16.	7.	0.	4.209270	-2.032597	1.695891
17.	6.	0.	3.905861	-0.691590	1.735539
18.	6.	0.	4.064550	-0.139980	0.275129
19.	6.	0.	2.717321	0.281261	-0.356601
20.	8.	0.	-0.758216	0.057690	-1.756719
21.	6.	0.	0.089663	1.362617	1.538901
22.	7.	0.	1.445018	1.369600	1.522590
23.	6.	0.	2.067699	1.557359	0.219948
24.	1.	0.	0.917522	1.614323	-1.648301
25.	8.	0.	-0.621451	1.213955	2.526924
26.	6.	0.	-3.926292	1.456948	0.320509
27.	6.	0.	-4.155771	0.800841	-1.018289
28.	6.	0.	-4.811552	-0.610739	-0.914600
29.	6.	0.	-4.005561	-1.558484	0.026200
30.	6.	0.	-2.508259	-1.731045	-0.398946
31.	6.	0.	-6.293753	-0.460096	-0.524375
32.	6.	0.	2.187979	4.043525	-1.591209
33.	6.	0.	0.137444	5.941335	-0.472128
34.	8.	0.	-2.288938	4.835570	-0.020762
35.	6.	0.	-2.241594	-3.217990	-0.246988
36.	6.	0.	-3.516819	-3.882968	-0.011319
37.	6.	0.	-4.520166	-2.996851	0.168315
38.	8.	0.	-1.164374	-3.803735	-0.275091
39.	8.	0.	-3.561447	-5.230489	0.103976
40.	6.	0.	-5.895303	-3.413338	0.595506
41.	1.	0.	-2.405539	-1.527915	-1.474156
42.	1.	0.	-4.031342	-1.105315	1.029249
43.	8.	0.	3.603227	-0.067521	2.738711
44.	8.	0.	4.889399	1.017221	0.249635
45.	1.	0.	-1.858320	2.536262	-1.119709
46.	1.	0.	-1.059364	3.990155	1.421877
47.	1.	0.	-2.820615	2.433876	1.786794
48.	1.	0.	-1.809454	-0.461447	1.269406
49.	1.	0.	-0.599754	-1.529954	0.576884
50.	1.	0.	5.974760	-4.779947	-1.614541
51.	1.	0.	5.167859	-4.527455	0.725586

52.	1.	0.	5.099122	-0.640279	-2.423249
53.	1.	0.	5.953810	-2.861829	-3.175488
54.	1.	0.	4.203087	-2.603122	2.529467
55.	1.	0.	2.020106	-0.562616	-0.317413
56.	1.	0.	2.931760	0.464792	-1.415565
57.	1.	0.	1.996506	1.145186	2.343772
58.	1.	0.	2.848131	2.317050	0.297198
59.	1.	0.	-4.734789	1.340318	1.042085
60.	1.	0.	-3.224849	0.739701	-1.585687
61.	1.	0.	-4.833820	1.422968	-1.620522
62.	1.	0.	-4.777920	-1.044705	-1.923831
63.	1.	0.	-6.766286	0.313688	-1.138335
64.	1.	0.	-6.417043	-0.166333	0.524272
65.	1.	0.	-6.859409	-1.379585	-0.675984
66.	1.	0.	2.584423	3.235363	-2.214609
67.	1.	0.	1.977174	4.887882	-2.251315
68.	1.	0.	2.997929	4.350087	-0.917372
69.	1.	0.	-0.635155	6.350371	-1.130502
70.	1.	0.	1.115488	6.266841	-0.827623
71.	1.	0.	-0.031054	6.395186	0.511734
72.	1.	0.	-3.096480	4.470458	0.367992
73.	1.	0.	-2.635729	-5.522465	0.014399
74.	1.	0.	-5.857169	-4.412411	1.038744
75.	1.	0.	-6.319763	-2.719470	1.327420
76.	1.	0.	-6.594140	-3.462673	-0.248250
77.	1.	0.	5.762472	0.780719	0.591458

**2-b**			Standard Orientation (Ångstroms)		
Center number	Atom number	Type	X	Y	Z
1.	6.	0.	0.966242	3.581718	-0.858888
2.	6.	0.	0.882184	2.075680	-0.673461
3.	6.	0.	-0.371125	1.553066	0.075494
4.	6.	0.	-1.593864	2.517321	-0.053319
5.	6.	0.	-1.197963	3.957408	0.325002
6.	6.	0.	0.046701	4.441374	-0.388059
7.	6.	0.	-2.818058	2.127440	0.754138
8.	6.	0.	-0.846223	0.214318	-0.553302
9.	6.	0.	-1.445579	-0.869150	0.328987
10.	6.	0.	5.453512	-3.916142	-1.252427
11.	6.	0.	5.032858	-3.735416	0.071753
12.	6.	0.	4.631362	-2.460681	0.444132
13.	6.	0.	4.626973	-1.385614	-0.455420

14.	6.	0.	5.042026	-1.578306	-1.763526
15.	6.	0.	5.464457	-2.855308	-2.159316
16.	7.	0.	4.189492	-2.010611	1.706772
17.	6.	0.	3.954537	-0.664611	1.709173
18.	6.	0.	4.082588	-0.155806	0.245949
19.	6.	0.	2.729562	0.274770	-0.381464
20.	8.	0.	-0.762762	0.064190	-1.760157
21.	6.	0.	0.112380	1.368331	1.528508
22.	7.	0.	1.469328	1.368769	1.503724
23.	6.	0.	2.083843	1.549978	0.195316
24.	1.	0.	0.921165	1.610897	-1.663542
25.	8.	0.	-0.589558	1.224707	2.522923
26.	6.	0.	-3.910227	1.474216	0.334427
27.	6.	0.	-4.150087	0.817672	-1.002242
28.	6.	0.	-4.811224	-0.591036	-0.893509
29.	6.	0.	-4.003486	-1.542161	0.042387
30.	6.	0.	-2.508999	-1.719988	-0.390173
31.	6.	0.	-6.290078	-0.433676	-0.493314
32.	6.	0.	2.199987	4.039360	-1.618003
33.	6.	0.	0.165913	5.942352	-0.491163
34.	8.	0.	-2.262291	4.847514	-0.020818
35.	6.	0.	-2.245902	-3.207542	-0.236621
36.	6.	0.	-3.522393	-3.868453	0.003355
37.	6.	0.	-4.522317	-2.979010	0.185993
38.	8.	0.	-1.170114	-3.795555	-0.266518
39.	8.	0.	-3.571429	-5.215628	0.119340
40.	6.	0.	-5.897427	-3.391380	0.617150
41.	1.	0.	-2.411639	-1.519379	-1.466327
42.	1.	0.	-4.022848	-1.089787	1.046013
43.	8.	0.	3.747055	0.040398	2.688841
44.	8.	0.	5.012949	0.922886	0.229432
45.	1.	0.	-1.846578	2.545026	-1.118755
46.	1.	0.	-1.028489	3.999667	1.416473
47.	1.	0.	-2.793672	2.450088	1.793092
48.	1.	0.	-1.796331	-0.451306	1.272500
49.	1.	0.	-0.594117	-1.524633	0.573225
50.	1.	0.	5.776825	-4.901527	-1.573652
51.	1.	0.	5.026917	-4.561725	0.775502
52.	1.	0.	5.052482	-0.750947	-2.466602
53.	1.	0.	5.800436	-3.020130	-3.177900
54.	1.	0.	4.221835	-2.549577	2.560819
55.	1.	0.	2.024011	-0.563374	-0.346327
56.	1.	0.	2.944566	0.460759	-1.440042
57.	1.	0.	2.018723	1.148564	2.326605

58.	1.	0.	2.863030	2.315403	0.257872
59.	1.	0.	-4.714950	1.361550	1.060791
60.	1.	0.	-3.222963	0.752403	-1.575320
61.	1.	0.	-4.829274	1.442202	-1.600644
62.	1.	0.	-4.786082	-1.025314	-1.902817
63.	1.	0.	-6.763307	0.342291	-1.103943
64.	1.	0.	-6.404909	-0.139628	0.556206
65.	1.	0.	-6.860805	-1.350567	-0.641216
66.	1.	0.	2.618739	3.218925	-2.209456
67.	1.	0.	1.974002	4.854122	-2.309974
68.	1.	0.	2.998432	4.391322	-0.952539
69.	1.	0.	-0.608593	6.351379	-1.147289
70.	1.	0.	1.143180	6.261798	-0.854381
71.	1.	0.	0.004613	6.401690	0.491328
72.	1.	0.	-3.069601	4.486312	0.372094
73.	1.	0.	-2.647375	-5.511692	0.026527
74.	1.	0.	-5.861362	-4.391706	1.057662
75.	1.	0.	-6.316730	-2.697905	1.352423
76.	1.	0.	-6.599461	-3.435978	-0.224207
77.	1.	0.	4.906550	1.391230	1.072642

**2-c**			Standard Orientation (Ångstroms)		
Center number	Atom number	Type	X	Y	Z
1.	6.	0.	1.669733	2.941411	-0.801531
2.	6.	0.	1.160391	1.511785	-0.868300
3.	6.	0.	-0.074528	1.184376	0.012685
4.	6.	0.	-0.938560	2.448109	0.329532
5.	6.	0.	-0.060676	3.592171	0.874038
6.	6.	0.	1.139006	3.883148	-0.003286
7.	6.	0.	-2.091404	2.221764	1.289313
8.	6.	0.	-1.031917	0.225566	-0.749475
9.	6.	0.	-1.807484	-0.840666	0.007419
10.	6.	0.	4.932821	-2.699720	2.840602
11.	6.	0.	5.679554	-1.828239	2.037187
12.	6.	0.	5.182874	-1.536726	0.772916
13.	6.	0.	3.972922	-2.076605	0.307018
14.	6.	0.	3.248378	-2.946001	1.109613
15.	6.	0.	3.734905	-3.256408	2.387806
16.	7.	0.	5.740713	-0.707810	-0.218257
17.	6.	0.	4.974465	-0.691913	-1.361041
18.	6.	0.	3.710986	-1.561846	-1.092043
19.	6.	0.	2.435774	-0.717161	-1.292585

20.	8.	0.	-1.173536	0.368722	-1.951239
21.	6.	0.	0.524721	0.527389	1.270537
22.	7.	0.	1.803707	0.177232	0.971915
23.	6.	0.	2.265035	0.507551	-0.370993
24.	1.	0.	0.917704	1.288854	-1.911649
25.	8.	0.	-0.035622	0.331386	2.341968
26.	6.	0.	-3.381930	2.019200	0.990300
27.	6.	0.	-4.002302	1.812246	-0.368934
28.	6.	0.	-5.049743	0.656916	-0.406408
29.	6.	0.	-4.461351	-0.678895	0.143116
30.	6.	0.	-3.178780	-1.155523	-0.619309
31.	6.	0.	-6.336941	1.110488	0.307023
32.	6.	0.	2.840397	3.227506	-1.730003
33.	6.	0.	1.677198	5.288485	0.101625
34.	8.	0.	-0.837659	4.789084	0.950870
35.	6.	0.	-3.384895	-2.648551	-0.808044
36.	6.	0.	-4.757097	-2.965183	-0.433204
37.	6.	0.	-5.383814	-1.904872	0.121197
38.	8.	0.	-2.567564	-3.486254	-1.173017
39.	8.	0.	-5.216475	-4.230750	-0.563765
40.	6.	0.	-6.740277	-2.011674	0.750781
41.	1.	0.	-3.174868	-0.733885	-1.634104
42.	1.	0.	-4.192186	-0.495477	1.194752
43.	8.	0.	5.228272	-0.127128	-2.409618
44.	8.	0.	3.716497	-2.652387	-2.008902
45.	1.	0.	-1.322822	2.798808	-0.634155
46.	1.	0.	0.285891	3.317848	1.887435
47.	1.	0.	-1.817572	2.255738	2.341958
48.	1.	0.	-1.866214	-0.601966	1.069419
49.	1.	0.	-1.182150	-1.744253	-0.078068
50.	1.	0.	5.297763	-2.945345	3.833239
51.	1.	0.	6.613462	-1.403109	2.391265
52.	1.	0.	2.321299	-3.382052	0.750525
53.	1.	0.	3.178209	-3.933683	3.027027
54.	1.	0.	6.651842	-0.274331	-0.170506
55.	1.	0.	1.570042	-1.383384	-1.212360
56.	1.	0.	2.474437	-0.358872	-2.328678
57.	1.	0.	2.353786	-0.349857	1.635458
58.	1.	0.	3.224533	1.031352	-0.300064
59.	1.	0.	-4.070066	1.952935	1.832877
60.	1.	0.	-3.233733	1.644645	-1.125850
61.	1.	0.	-4.528078	2.728587	-0.674271
62.	1.	0.	-5.298153	0.495628	-1.464708
63.	1.	0.	-6.619830	2.112546	-0.031641

64.	1.	0.	-6.212505	1.155854	1.394898
65.	1.	0.	-7.180870	0.452028	0.100688
66.	1.	0.	3.164547	2.330587	-2.264785
67.	1.	0.	2.573714	3.974054	-2.486333
68.	1.	0.	3.715005	3.612454	-1.194493
69.	1.	0.	0.947288	6.010236	-0.277457
70.	1.	0.	2.612298	5.418464	-0.444286
71.	1.	0.	1.851657	5.555326	1.150911
72.	1.	0.	-1.649780	4.567484	1.428414
73.	1.	0.	-4.459831	-4.743028	-0.903133
74.	1.	0.	-6.957560	-3.058285	0.981960
75.	1.	0.	-6.807309	-1.426552	1.673032
76.	1.	0.	-7.536006	-1.658325	0.083915
77.	1.	0.	3.829058	-2.267121	-2.892539

**2-d**			Standard Orientation (Ångstroms)		
Center number	Atom number	Type	X	Y	Z
1.	6.	0.	1.002560	3.453683	-0.912039
2.	6.	0.	0.878563	1.958630	-0.662041
3.	6.	0.	-0.398423	1.484378	0.085537
4.	6.	0.	-1.570991	2.515719	-0.015525
5.	6.	0.	-1.071564	3.912524	0.403791
6.	6.	0.	0.155320	4.355883	-0.382627
7.	6.	0.	-2.824422	2.176111	0.767795
8.	6.	0.	-0.931451	0.177602	-0.560839
9.	6.	0.	-1.559835	-0.896949	0.310202
10.	6.	0.	5.821569	-3.776958	-1.242811
11.	6.	0.	5.373665	-3.648744	0.078616
12.	6.	0.	4.857280	-2.418313	0.462219
13.	6.	0.	4.769078	-1.339037	-0.428164
14.	6.	0.	5.220034	-1.477385	-1.732119
15.	6.	0.	5.753732	-2.708384	-2.138689
16.	7.	0.	4.371886	-2.018961	1.720236
17.	6.	0.	4.005758	-0.693366	1.745122
18.	6.	0.	4.134150	-0.152478	0.277953
19.	6.	0.	2.767441	0.209271	-0.346960
20.	8.	0.	-0.860255	0.044001	-1.770999
21.	6.	0.	0.070590	1.258707	1.538633
22.	7.	0.	1.424975	1.219912	1.523947
23.	6.	0.	2.065856	1.452574	0.239481
24.	1.	0.	0.919202	1.452667	-1.631511
25.	8.	0.	-0.645056	1.120855	2.523851

26.	6.	0.	-3.923068	1.544917	0.335903
27.	6.	0.	-4.177389	0.898800	-1.004510
28.	6.	0.	-4.899222	-0.480822	-0.912069
29.	6.	0.	-4.136053	-1.472739	0.017248
30.	6.	0.	-2.649612	-1.707745	-0.413221
31.	6.	0.	-6.370872	-0.264525	-0.513464
32.	6.	0.	2.166630	3.802463	-1.812742
33.	6.	0.	0.313679	5.855609	-0.487926
34.	8.	0.	-2.134733	4.862325	0.295584
35.	6.	0.	-2.444302	-3.204246	-0.262624
36.	6.	0.	-3.746346	-3.815659	-0.029105
37.	6.	0.	-4.712181	-2.888540	0.150425
38.	8.	0.	-1.392197	-3.834217	-0.293373
39.	8.	0.	-3.847640	-5.160850	0.081617
40.	6.	0.	-6.105347	-3.248710	0.570280
41.	1.	0.	-2.543030	-1.508937	-1.488907
42.	1.	0.	-4.139385	-1.026329	1.023598
43.	8.	0.	3.673913	-0.072389	2.740829
44.	8.	0.	4.909844	1.038392	0.233119
45.	1.	0.	-1.809971	2.573256	-1.086409
46.	1.	0.	-0.843734	3.899461	1.478532
47.	1.	0.	-2.822705	2.538122	1.791259
48.	1.	0.	-1.896634	-0.477692	1.258023
49.	1.	0.	-0.726595	-1.578667	0.546183
50.	1.	0.	6.231283	-4.727480	-1.570651
51.	1.	0.	5.432656	-4.481016	0.772926
52.	1.	0.	5.168465	-0.640707	-2.422837
53.	1.	0.	6.113680	-2.832140	-3.154886
54.	1.	0.	4.392416	-2.580210	2.559872
55.	1.	0.	2.105506	-0.662676	-0.306054
56.	1.	0.	2.970777	0.405361	-1.405826
57.	1.	0.	1.970578	1.010934	2.352811
58.	1.	0.	2.819126	2.235337	0.352739
59.	1.	0.	-4.743957	1.472262	1.048741
60.	1.	0.	-3.251181	0.795085	-1.573168
61.	1.	0.	-4.826266	1.552527	-1.606664
62.	1.	0.	-4.890488	-0.907369	-1.925209
63.	1.	0.	-6.809419	0.535727	-1.118719
64.	1.	0.	-6.474204	0.026698	0.537794
65.	1.	0.	-6.979356	-1.155631	-0.669332
66.	1.	0.	2.267157	4.872650	-1.991906
67.	1.	0.	3.122542	3.440451	-1.415075
68.	1.	0.	2.034418	3.316913	-2.789035
69.	1.	0.	-0.415618	6.299620	-1.174888

70.	1.	0.	1.310386	6.151879	-0.815774
71.	1.	0.	0.125517	6.318295	0.486584
72.	1.	0.	-2.472137	4.819528	-0.610396
73.	1.	0.	-2.934730	-5.490706	-0.007591
74.	1.	0.	-6.112387	-4.253058	1.003047
75.	1.	0.	-6.501122	-2.544569	1.308458
76.	1.	0.	-6.803369	-3.258135	-0.275596
77.	1.	0.	5.794587	0.843334	0.571164

**2-e**			Standard Orientation (Ångstroms)		
Center number	Atom number	Type	X	Y	Z
1.	6.	0.	0.972708	3.600860	-0.856198
2.	6.	0.	0.873269	2.094832	-0.685415
3.	6.	0.	-0.383823	1.582424	0.066066
4.	6.	0.	-1.603776	2.554246	-0.062849
5.	6.	0.	-1.187506	3.979008	0.351051
6.	6.	0.	0.068828	4.460736	-0.354376
7.	6.	0.	-2.851899	2.162732	0.706425
8.	6.	0.	-0.849953	0.236257	-0.551751
9.	6.	0.	-1.432060	-0.845944	0.341670
10.	6.	0.	5.360398	-3.962771	-1.249281
11.	6.	0.	4.949109	-3.765291	0.075422
12.	6.	0.	4.568521	-2.481796	0.439498
13.	6.	0.	4.575742	-1.413892	-0.468584
14.	6.	0.	4.981095	-1.623228	-1.777191
15.	6.	0.	5.382597	-2.909463	-2.164718
16.	7.	0.	4.140139	-2.015228	1.700837
17.	6.	0.	3.926427	-0.665857	1.693773
18.	6.	0.	4.051536	-0.171228	0.225585
19.	6.	0.	2.699408	0.269649	-0.396626
20.	8.	0.	-0.763750	0.078128	-1.757830
21.	6.	0.	0.100611	1.406987	1.520150
22.	7.	0.	1.458318	1.384473	1.490142
23.	6.	0.	2.071345	1.553937	0.179695
24.	1.	0.	0.905614	1.634791	-1.677807
25.	8.	0.	-0.600321	1.288408	2.517228
26.	6.	0.	-3.909940	1.465992	0.274365
27.	6.	0.	-4.114379	0.777858	-1.053238
28.	6.	0.	-4.769859	-0.632433	-0.934687
29.	6.	0.	-3.973404	-1.559635	0.032827
30.	6.	0.	-2.470550	-1.730731	-0.370187
31.	6.	0.	-6.256913	-0.478566	-0.564888

32.	6.	0.	2.203171	4.058327	-1.620056
33.	6.	0.	0.220673	5.963060	-0.412515
34.	8.	0.	-2.278324	4.882507	0.153638
35.	6.	0.	-2.192254	-3.210067	-0.172760
36.	6.	0.	-3.465767	-3.880281	0.055907
37.	6.	0.	-4.479542	-2.998543	0.195879
38.	8.	0.	-1.108675	-3.784988	-0.166747
39.	8.	0.	-3.502158	-5.225381	0.201566
40.	6.	0.	-5.860024	-3.416254	0.603576
41.	1.	0.	-2.359583	-1.557352	-1.449782
42.	1.	0.	-4.014141	-1.087413	1.026462
43.	8.	0.	3.737877	0.051234	2.668462
44.	8.	0.	4.992631	0.897759	0.194176
45.	1.	0.	-1.828980	2.586830	-1.137914
46.	1.	0.	-1.029817	3.997361	1.438238
47.	1.	0.	-2.885609	2.545726	1.721955
48.	1.	0.	-1.799928	-0.418905	1.274611
49.	1.	0.	-0.568836	-1.478875	0.604398
50.	1.	0.	5.667342	-4.955402	-1.564176
51.	1.	0.	4.934082	-4.585828	0.785732
52.	1.	0.	5.000097	-0.801751	-2.486973
53.	1.	0.	5.711006	-3.087436	-3.183563
54.	1.	0.	4.168686	-2.548069	2.558863
55.	1.	0.	1.986053	-0.561230	-0.353914
56.	1.	0.	2.910793	0.448861	-1.457130
57.	1.	0.	2.007949	1.169278	2.313999
58.	1.	0.	2.861037	2.308916	0.237992
59.	1.	0.	-4.735370	1.366523	0.978729
60.	1.	0.	-3.177005	0.705952	-1.608357
61.	1.	0.	-4.786074	1.385429	-1.678064
62.	1.	0.	-4.723286	-1.084707	-1.935506
63.	1.	0.	-6.723210	0.284276	-1.197061
64.	1.	0.	-6.392960	-0.167212	0.476914
65.	1.	0.	-6.819224	-1.401564	-0.708296
66.	1.	0.	2.594976	3.249956	-2.245670
67.	1.	0.	1.984278	4.902195	-2.278743
68.	1.	0.	3.019200	4.369670	-0.955633
69.	1.	0.	-0.485169	6.420603	-1.115045
70.	1.	0.	1.228933	6.269377	-0.693884
71.	1.	0.	-0.007159	6.400362	0.565754
72.	1.	0.	-2.536371	4.827172	-0.777465
73.	1.	0.	-2.573024	-5.512271	0.135221
74.	1.	0.	-5.825507	-4.408312	1.062486
75.	1.	0.	-6.301709	-2.713393	1.316459

76.	1.	0.	-6.542190	-3.481213	-0.252681
77.	1.	0.	4.904926	1.366040	1.039631

GTATAGACCTACCTGATCCGAGGTCACCTGGTTAAGATTGATGGTGTTCGCCGGCGGGCGCCGGCCGGGCCTA
CAGAGCGGGTGACGAAGCCCCATACGCTCGAGGACCGGACGCGGTGCCGCCGCTGCCTTTCGGGCCCGCCC
CCCGGAAGCGGGGGGCGAGAGCCCAACACACAAGCCGTGCTTGAGGGCAGCAATGACGCTCGGACAGGCA
TGCCCCCCGGAATACCAGGGGGCGCAATGTGCGTTCAAAGACTCGATGATTCACTGAATTCTGCAATTCACAT
TACTTATCGCATTTCGCTGCGTTCTTCATCGATGCCGGAACCAAGAGATCCGTTGTTGAAAGTTTTAACTGATT
TAGTCAAGTACTCAGACTGCAATCTTCAGACAAGAGTTCGTTTGTGTGTCTTCGGCGGGCGCGGGCCCGGGG
GCGGATGCCCCCCGGCGGCCGTGAGGCGGGCCCGCCGAAGCAACAAGGTACGATAAACACGGGTGGGAGG
TTGGACCCAGAGGGCCCTCACTCGGTAATGATCCTT

Figure S25. 18S rDNA gene sequence of 375

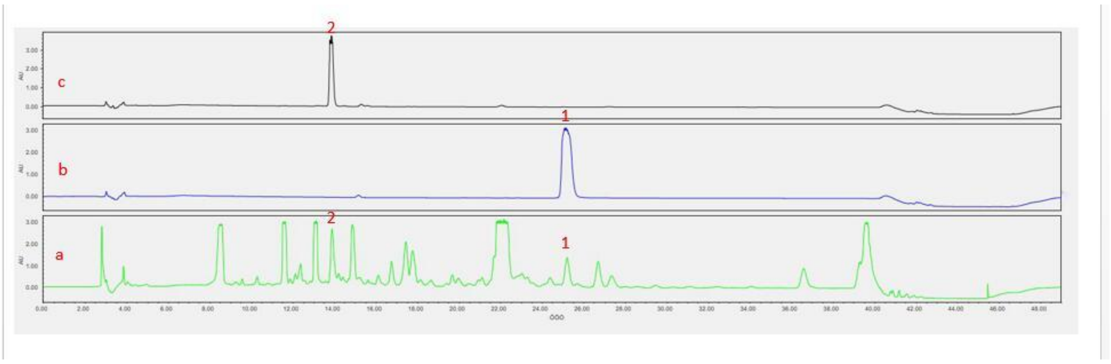

Figure S26. HPLC chromatogram analysis of the crude extracts from 375 and pure compounds **1** and **2**

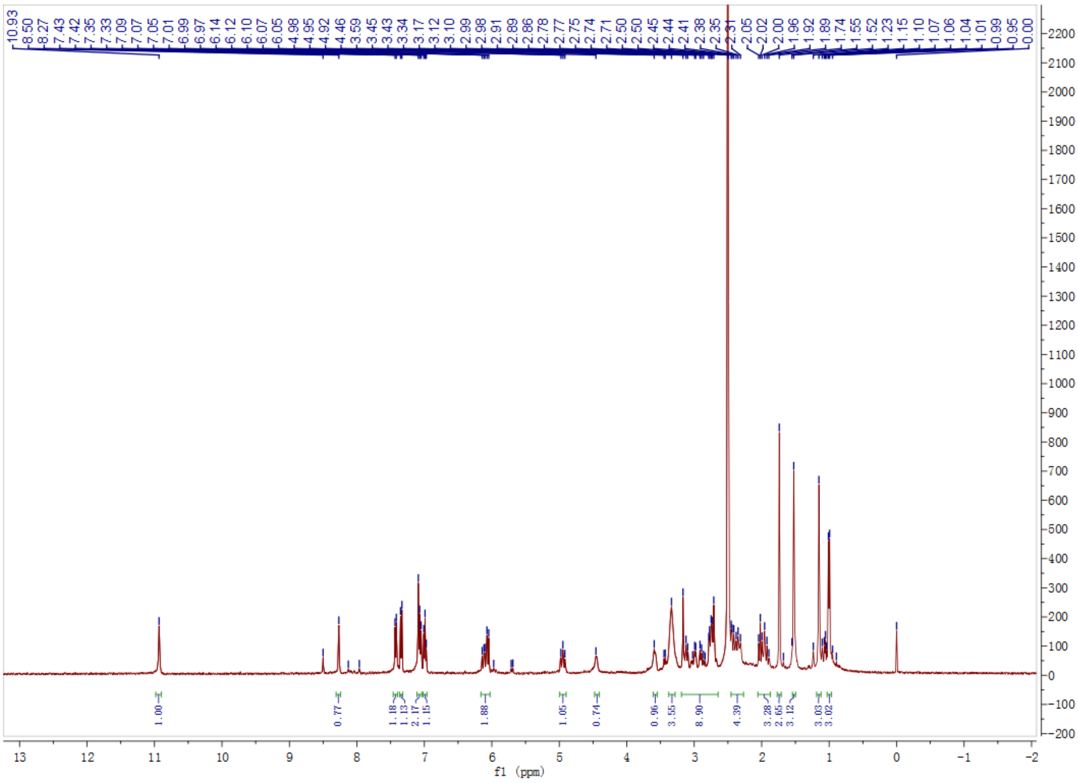

Figure S27. ^{1}H NMR spectrum of compound **3** in DMSO-d_6 (400 MHz)

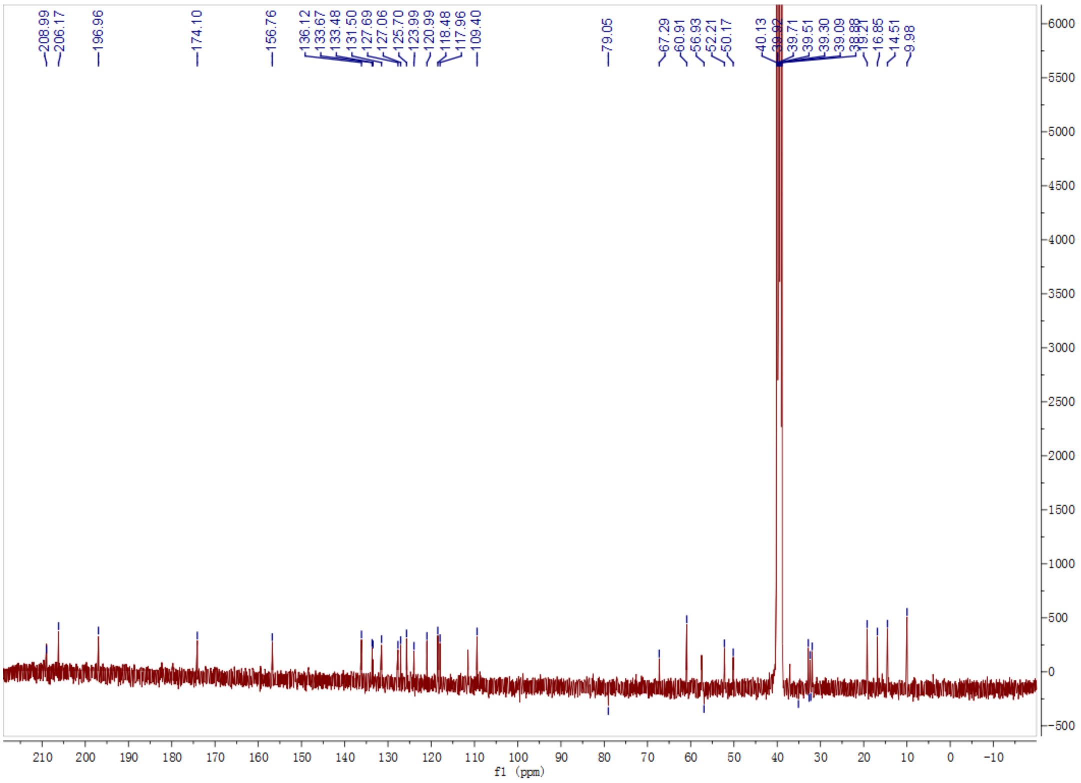

Figure S28. ^{13}C NMR spectrum of compound **3** in DMSO-d_6 (100 MHz)

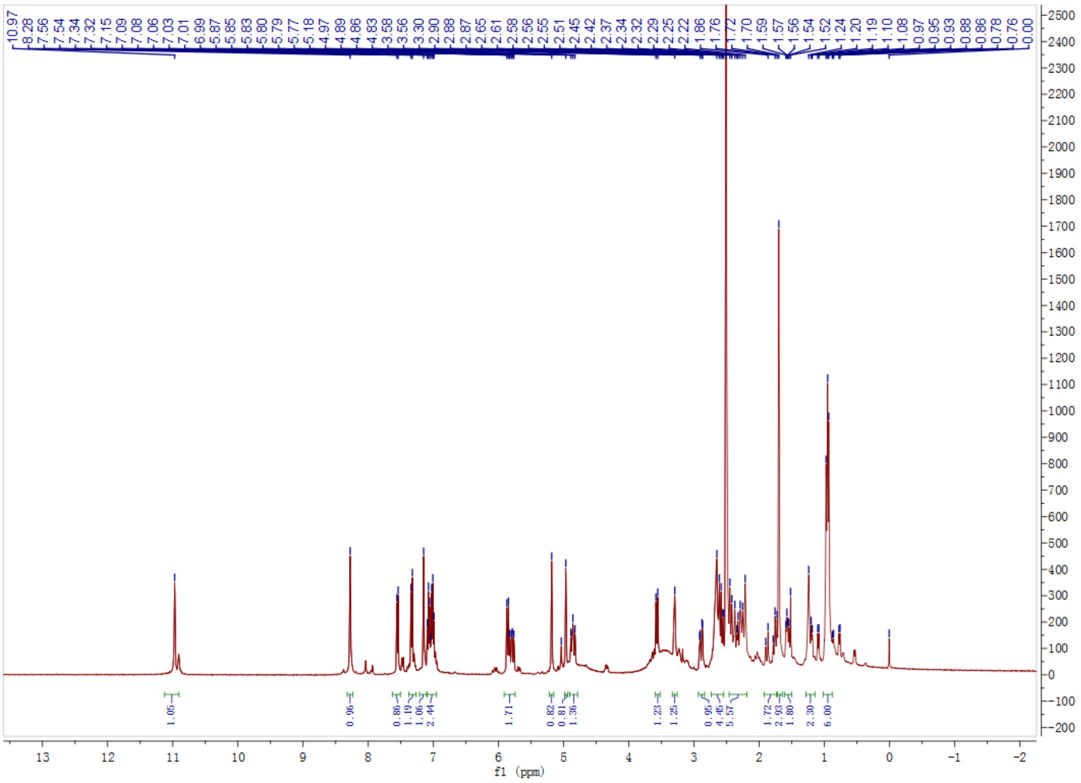

Figure S29. ^{1}H NMR spectrum of compound **4** in DMSO-d_6 (400 MHz)

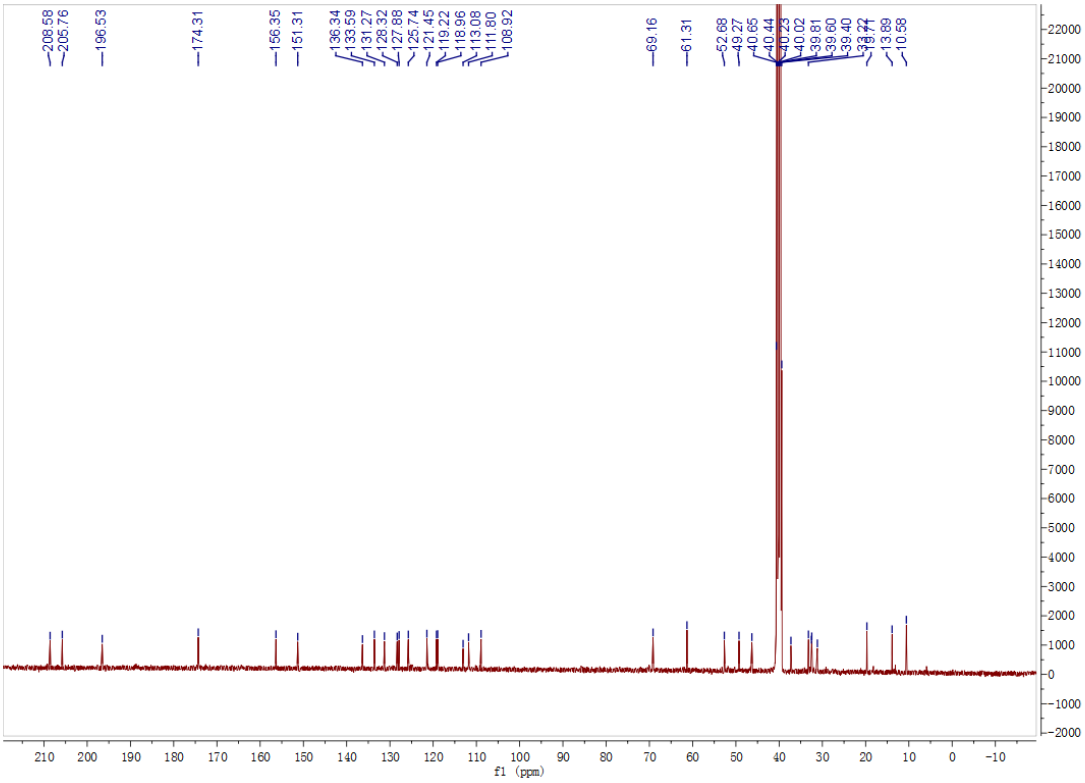

Figure S30. ^{13}C NMR spectrum of compound **4** in DMSO-d_6 (100 MHz)

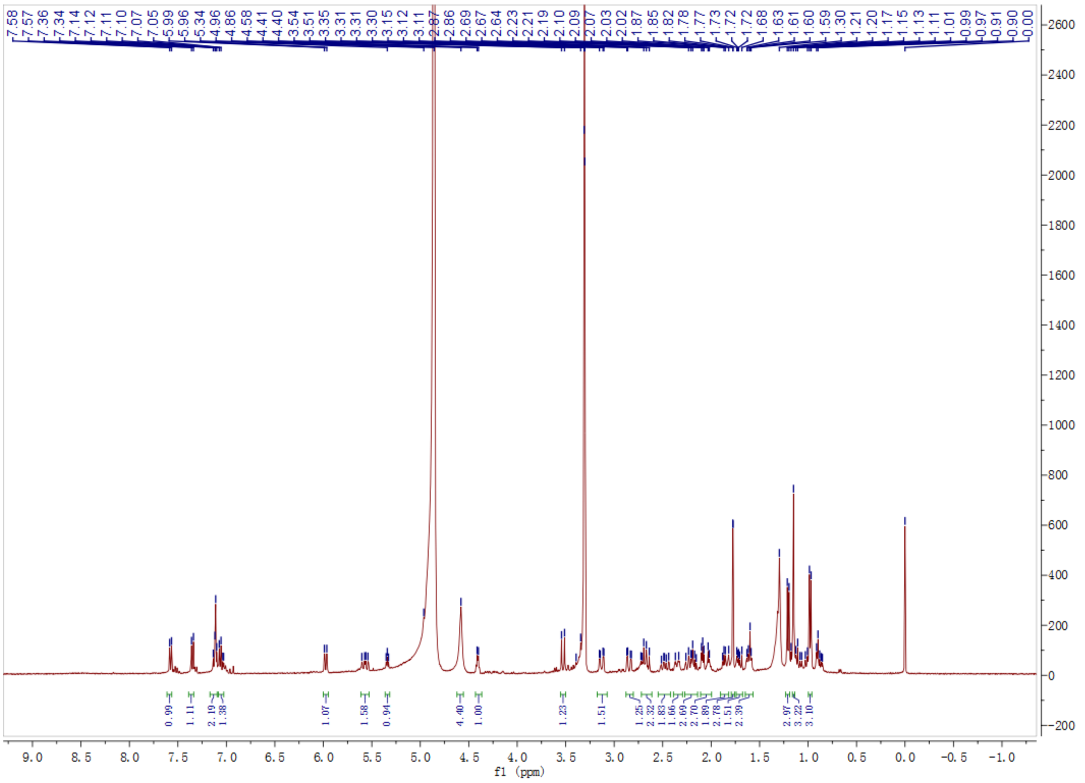

Figure S31. ^{1}H NMR spectrum of compound **5** in CD_3OD (400 MHz)

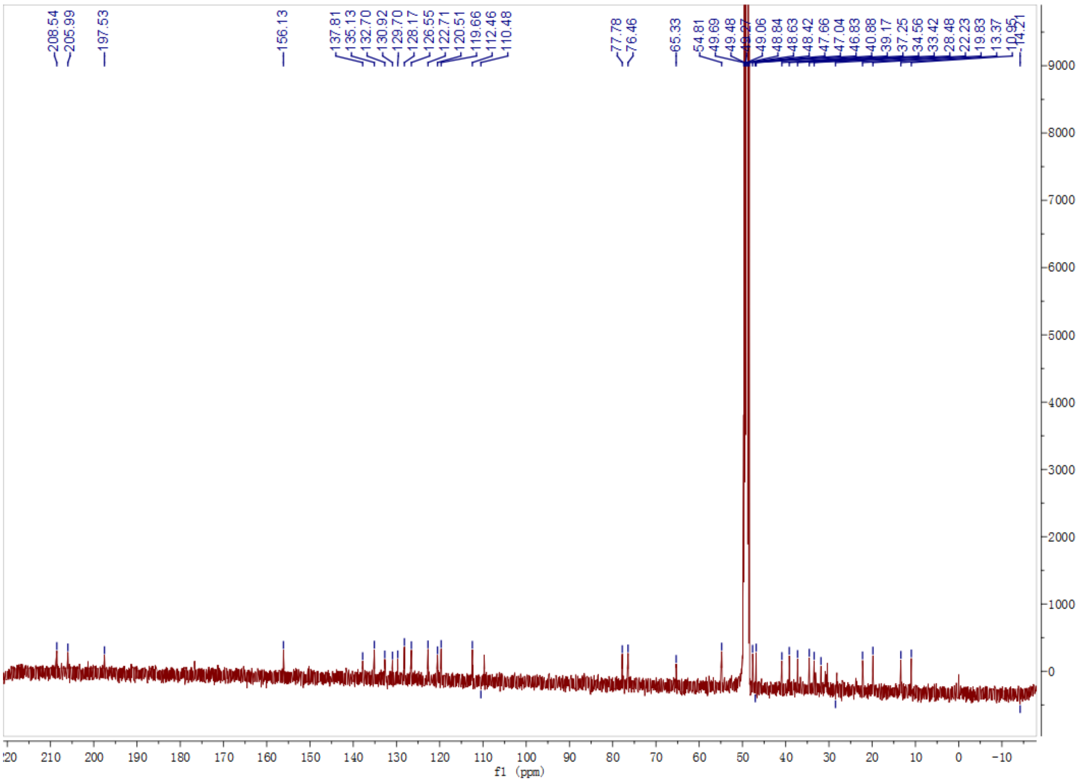

Figure S32. ^{13}C NMR spectrum of compound **5** in **C**D$_3$OD (100 MHz)

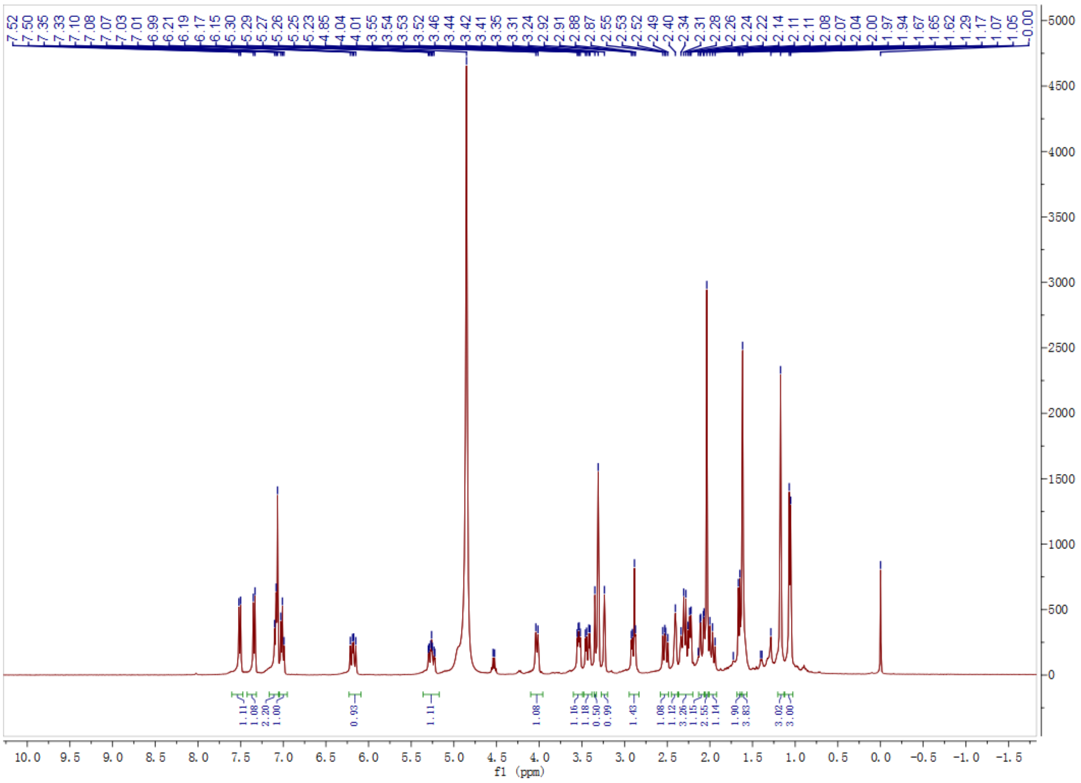

Figure S33. ^{1}H NMR spectrum of compound **6** in **C**D$_3$OD (400 MHz)

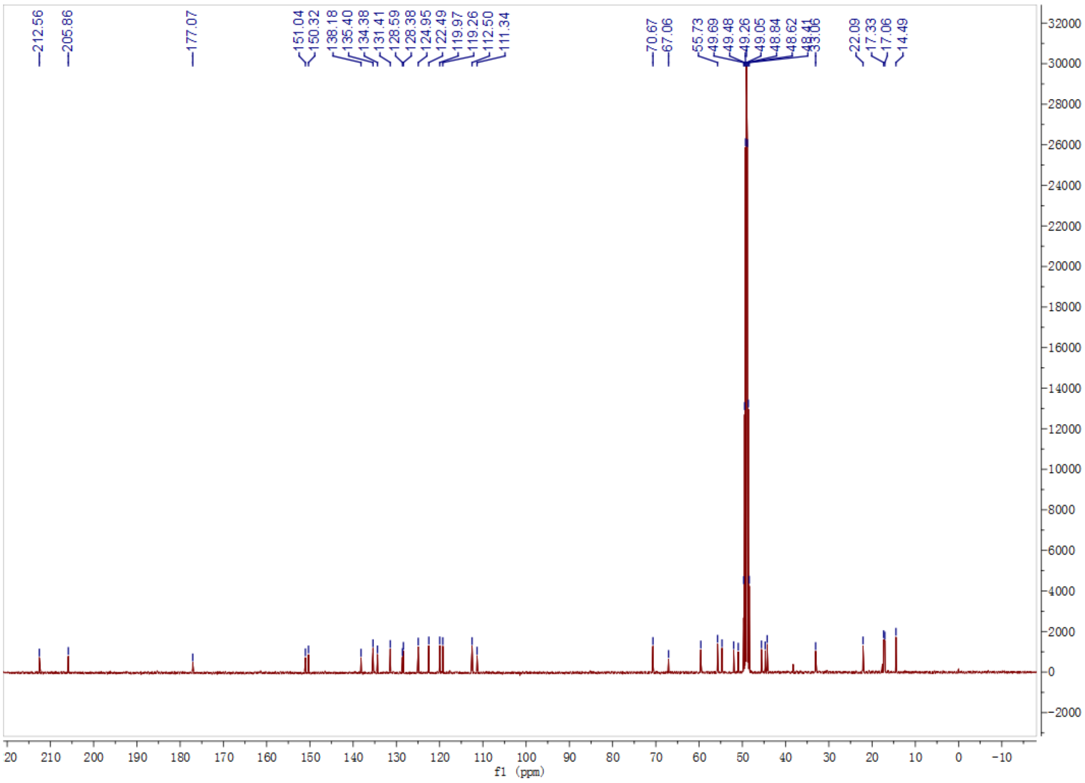

Figure S34. ^{13}C NMR spectrum of compound **6** in **C**D_3OD (100 MHz)

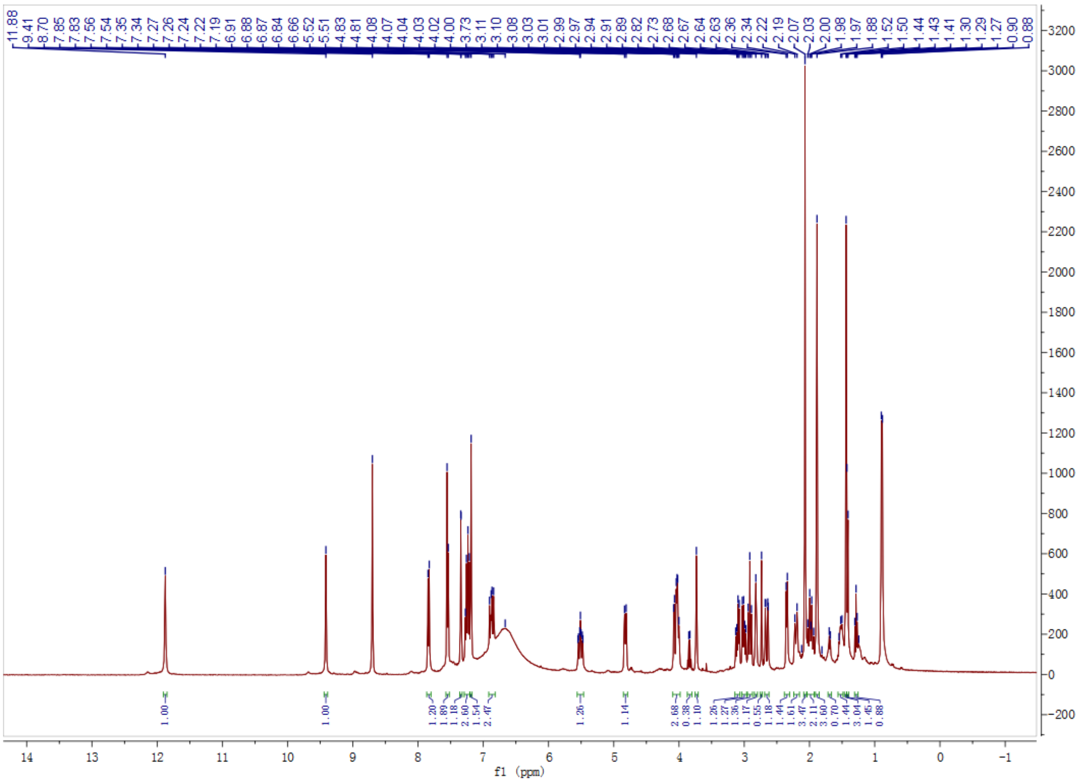

Figure S35. ^{1}H NMR spectrum of compound **6** in C_5D_5N (400 MHz)

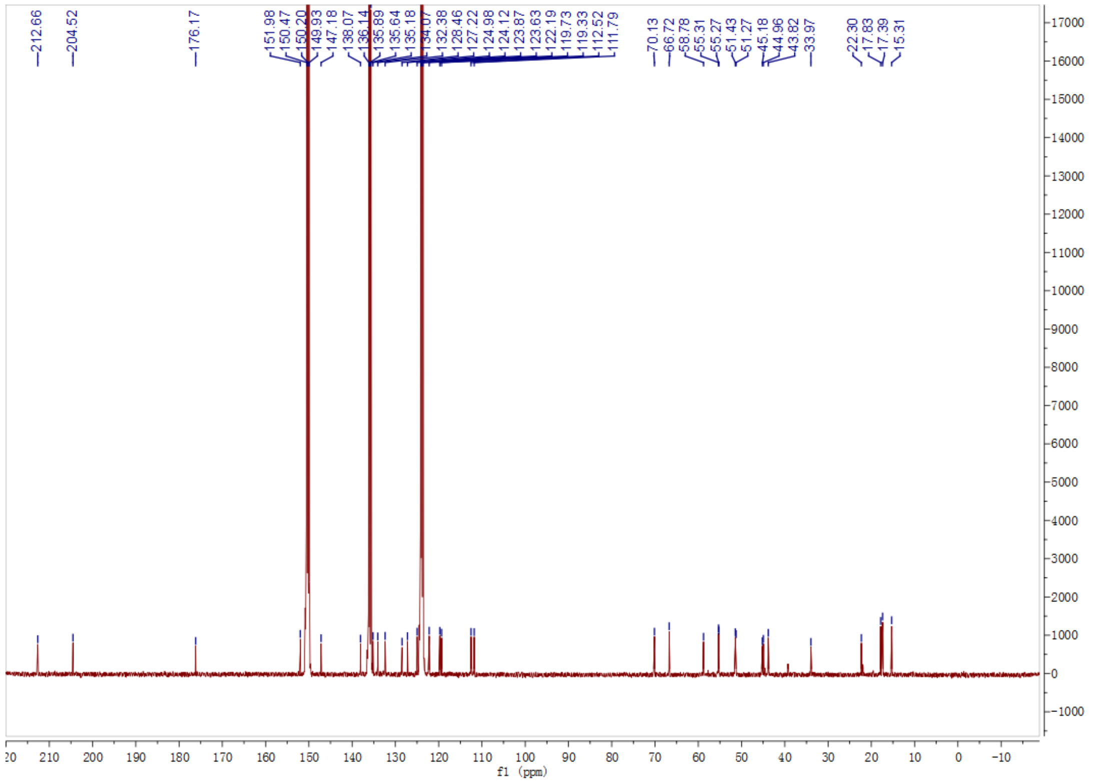

Figure S36. ^{13}C NMR spectrum of compound **6** in C_5D_5N (100 MHz)

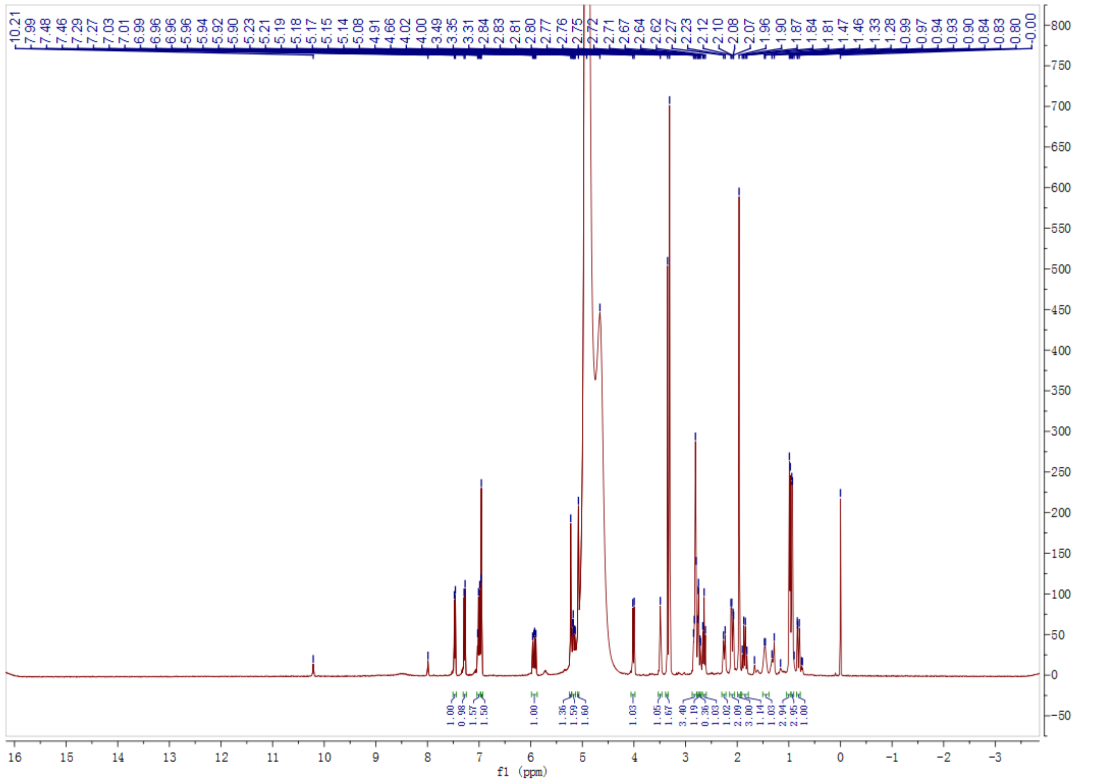

Figure S37. ^{1}H NMR spectrum of compound **7** in CD_3OD (400 MHz)

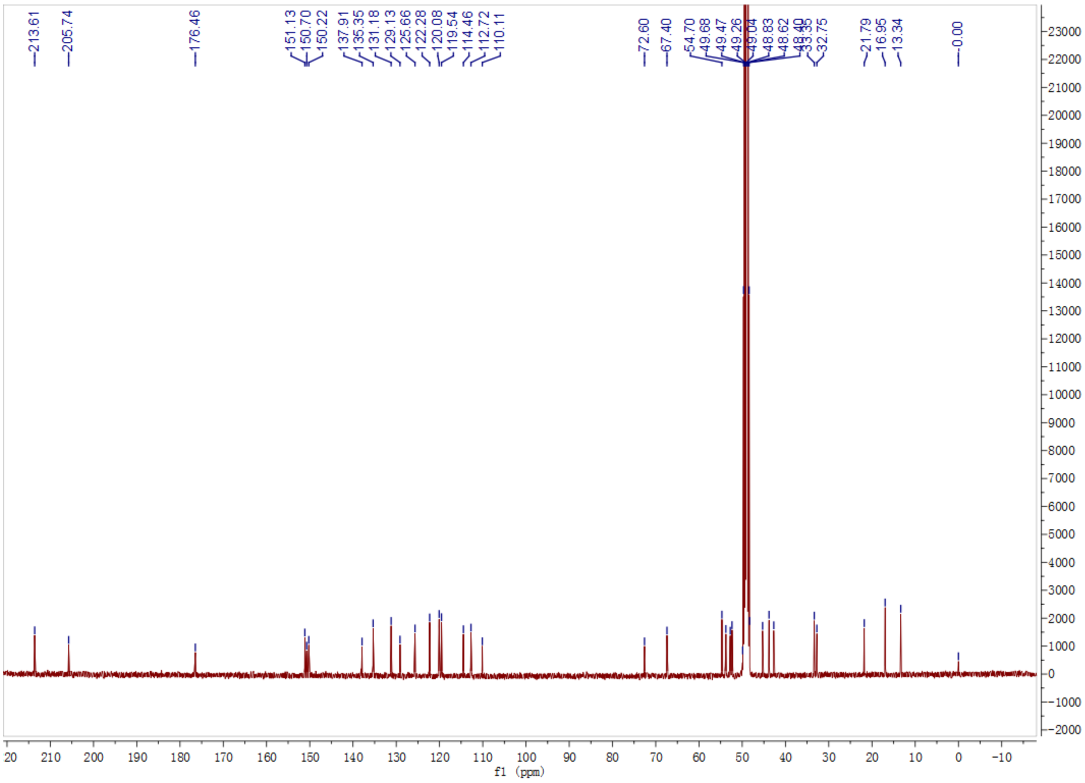

Figure S38. ^{13}C NMR spectrum of compound **7** in CD_3OD (100 MHz)

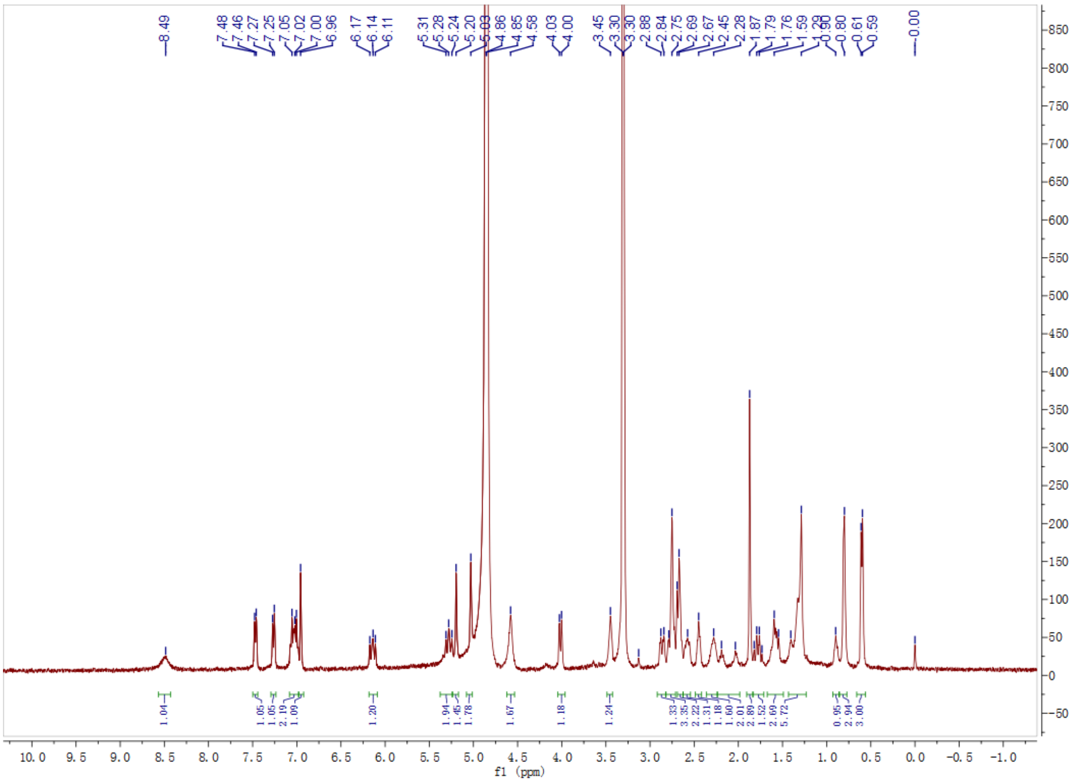

Figure S39. ^{1}H NMR spectrum of compound **8** in CD_3OD (400 MHz)

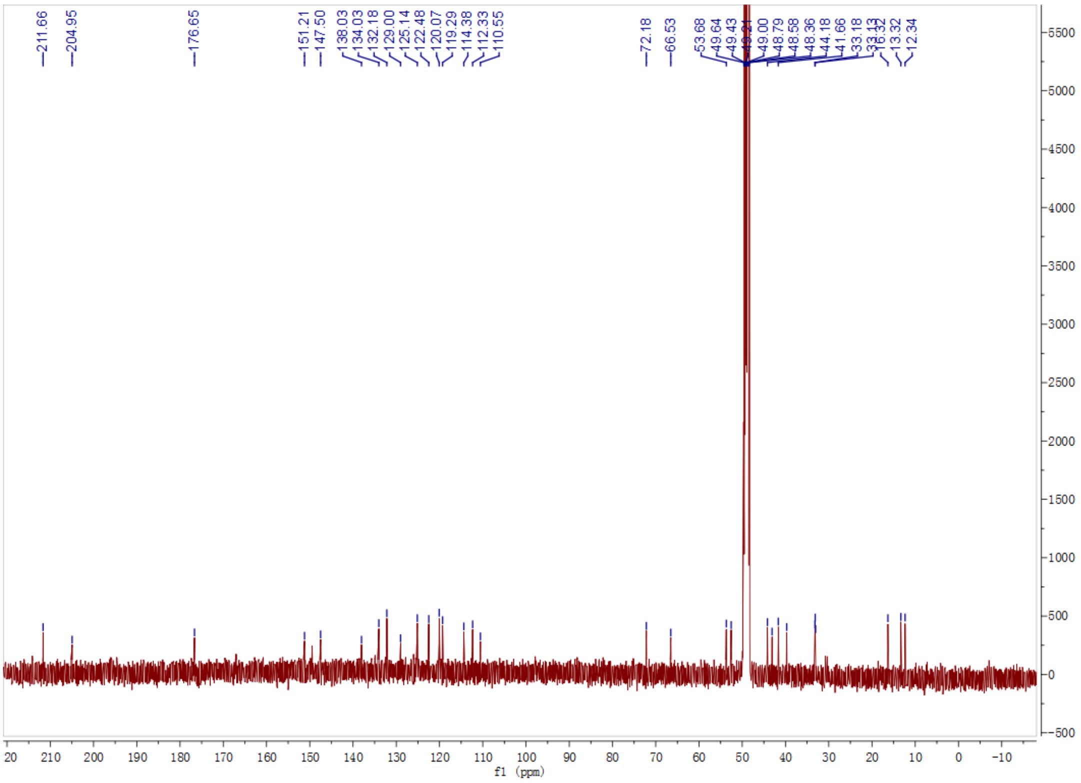

Figure S40. ^{13}C NMR spectrum of compound **8** in CD_3OD (100 MHz)
